# A Tunable Strategy for Continuous Production of Electrolyte‐Free Formic Acid and Sodium Formate in a Solid‐State‐Electrolyte Based Electrocatalytic CO_2_ Reduction System

**DOI:** 10.1002/advs.202508152

**Published:** 2025-06-30

**Authors:** Jinrui Guo, Wenqiang Qi, Rongrong Mo, Feiyi Yuan, Lin Wang, Yongmei Li

**Affiliations:** ^1^ State Key Laboratory of Water Pollution Control and Green Resource Recycling College of Environmental Science and Engineering Tongji University Shanghai 200092 P. R. China; ^2^ Shanghai Institute of Pollution Control and Ecological Security Shanghai 200092 P. R. China

**Keywords:** electrochemical CO_2_ reduction, electrolyte‐free formate production, electrolyte‐free formic acid solution production, solid‐state‐electrolyte

## Abstract

Electrocatalytic CO_2_ reduction reaction (CO_2_RR) based on solid‐state‐electrolyte (SSE) reactors can efficiently convert CO_2_ to electrolyte‐free formic acid (HCOOH) solution, thereby circumventing energy‐intensive downstream separation processes and further fostering the advancement of carbon‐neutral technologies. However, the absence of alkali metal cations in the SSE‐based CO_2_RR process at the cathode poses a challenge, constraining the performance and stability of CO_2_RR and exacerbating the hydrogen evolution side reaction. Herein, a novel strategy for the tunable production of both electrolyte‐free HCOOH and sodium formate (HCOONa) solution through the regulation of anolyte composition in an SSE‐based cell is reported. Employing this strategy, the continuous generation of a ≈0.27 m electrolyte‐free HCOONa solution and ≈0.22 m electrolyte‐free HCOOH solution with extended stabilities of 300 and 200 h, respectively is achieved. More importantly, the introduction of sodium ions resulted in a reduction of cell voltage by ≈1000 mV and further enhances the stability of the cell. In situ infrared spectroscopy and density functional theory calculations reveal that GB‐Bi requires a lower applied potential for formate production, owing to its stronger binding energy to the key intermediate OCHO* compared to Bi. Finally, a techno‐economic analysis indicates that this strategy for HCOONa solution production possesses excellent economic viability.

## Graphical abstract

A tunable strategy based on Membrane electrode assembly‐SSE (MEA‐SSE) cells to continuously generate electrolyte‐free formic acid/sodium formate solution, holds high potential for industrial applications.

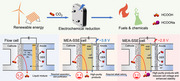

## 1 Introduction

The extensive utilization of fossil fuels, driven by societal progress, has led to an energy crisis and climate change, posing a significant threat to global ecosystems.^[^ ^1^ ^]^ In the context of climate change mitigation and the development of new carbon resources, electrocatalytic carbon dioxide reduction (CO_2_RR) to reduce CO_2_ emission and convert it into valuable chemicals is imperative.^[^ ^2^ ^]^ Over the past decade, a variety of catalysts have been engineered for the CO_2_RR, yielding products from C1 to C3, including carbon monoxide (CO),^[^ ^3^ ^]^ formic acid/formate (HCOOH/HCOO^−^),^[^ ^4^ ^]^ methane (CH_4_),^[^ ^5^ ^]^ ethylene (C_2_H_4_),^[^ ^2^, ^6^ ^]^ acetic acid/acetate (CH_3_COOH/CH_3_COO^−^),^[^ ^7^ ^]^ ethanol (C_2_H_5_OH),^[^ ^8^ ^]^ n‐propanol (C_3_H_8_O),^[^ ^9^ ^]^ etc. Notably, HCOOH and HCOO^−^, resulting from the two‐electron reduction of CO_2_, stands out among CO_2_RR products for its promising economic potential, high volumetric hydrogen storage capacity, and favorable storage and transportation characteristics.^[^ ^10^ ^]^

Currently, significant research is focused on achieving high current density (exceeding 200 mA cm^−2^), high product selectivity (above 90% of faradaic efficiency (FE)), and long‐time stability (above 100 h) to meet the criteria for industrial viability.^[^ ^2^, ^4^, ^11^ ^]^ For example, Liu et al. devised SnO_2_ assisted by surface hydroxyls that can operate at a high current density of 200 mA cm^−2^ for at least 6 h while maintaining FE of formic acid over 80%.^[^ ^12^ ^]^ Similarly, Ma et al. synthesized a series of sulfur‐modified Cu_2_O for CO_2_RR to produce HCOOH, achieving a partial current density of 260 ± 16 mA cm^−2^ and FE of ≈70% in a flow cell system.^[^ ^13^ ^]^ Among these catalysts, bismuth (Bi) based catalysts are widely favored because of their superior catalytic performance, good stability, safety, and non‐toxicity.^[^ ^14^ ^]^ Representatively, Wang et al. reconstructed Bi_19_Cl_3_S_27_ under negative potential to prepare vacancy‐rich defects Bi nanosheets, which achieved a remarkable HCOOH partial current density of 400 mA cm^−2^ with 96% FE_HCOOH_.^[^ ^15^ ^]^

Despite recent progress, liquid HCOOH/HCOO^−^ products, present in the form of ions in liquid electrolytes, necessitating impurity‐ion separation and acidification processes (such as reverse osmosis and electrodialysis) before the distillation purification process. These processes lead to liquid separation costs that exceed 80% of the total production costs, making the CO_2_RR to HCOOH products route not yet economically viable.^[^ ^16^ ^]^ In addition, the use of high‐concentration electrolytes can facilitate the corrosion of gas diffusion electrodes, which in turn inducing flooding and salt precipitation.^[^ ^4^, ^17^ ^]^ These challenges compromise the efficiency of CO_2_ utilization and reduce the stability of CO_2_RR, with the stability of the catalyst typically not exceeding 100 h. To address this issue, Yang et al. pioneered a three‐compartment cell design, employing cation ion exchange resin in the middle chamber to replace the liquid electrolyte, and the produced HCOOH was efficiently released via the deionized water.^[^ ^18^ ^]^ Subsequently, Xia et al. introduced a solid‐state electrolyte (SSE) in the middle chamber to decouple the ion‐conduction and product‐collection functions of the liquid electrolyte, successfully yielding a 0.11 mol L^−1^ (M) formic acid solution continuously in 100 h.^[^ ^19^ ^]^ Building on this foundation, they further proposed an all‐solid‐state electrolysis cell that applied humidified N_2_ flow to release products, which successfully generated a 0.35 m formic acid solution in 30 h.^[^ ^20^ ^]^ However, these advancements are accompanied by challenges such as low current density and catalytic activity. Despite employing costly hydrogen at the anode to reduce the overpotential of the reaction, the stable operation of this system is limited to 30 mA cm^−2^ for up to 30 h.^[^ ^20^ ^]^ This suggests that while the strategy of replacing liquid electrolytes with SSE has made significant progress in HCOOH production, there remains considerable potential for further improvement of efficiency and cost‐effectiveness.

Additionally, recent research has demonstrated that alkali metal cations, such as potassium (K^+^) and sodium (Na^+^), can significantly promote CO_2_RR by inhibiting the migration of hydronium ions (H^+^) and stabilizing crucial intermediates, thereby playing a critical role in CO_2_RR.^[^ ^21^ ^]^ Conversely, the absence of metal cation catalysis in CO_2_RR results in diminished catalytic efficiency, decreased current density, and pronounced hydrogen evolution side reactions.^[^ ^16^, ^22^ ^]^ In the conventional SSE‐based cell configuration, the protons generated by oxygen evolution reaction (OER) on the anode side migrate through the cation exchange membrane (CEM) toward the SSE layer, coupling with HCOO^−^ to form HCOOH.^[^ ^19^ ^]^ However, the cathode lacking alkali metal cations limits CO_2_RR performance and promotes severe side reaction of hydrogen evolution reaction (HER). Therefore, integrating alkali metal cations into an SSE‐based cell exhibits significant application potential by enhancing CO_2_RR performance and regulating product composition.

In this work, we explored a novel tunable process for producing both electrolyte‐free HCOOH solution and electrolyte‐free sodium formate (HCOONa) solution by regulating the anolyte composition. First, we synthesized a grain boundary enriched Bi catalyst (GB‐Bi) via a straightforward chemical reduction process. High‐resolution transmission electron microscopy (HR‐TEM) revealed distinct lattice orientations and interplanar distance stretch in GB‐Bi, conforming the formation of diverse GBs. The GB‐Bi exhibited exceptional catalytic performance in three‐electrode flow cells, achieving high HCOO^−^ FEs over a broad current density range. In Membrane electrode assembly‐SSE (MEA‐SSE) cell configuration, the GB‐Bi catalyst enabled the industrial‐scale, steady, and continuous production of electrolyte‐free HCOOH solutions. By simply adjusting the anolyte composition, we could readily achieve electrolyte‐free HCOONa production, which exhibited enhanced stability and reduced cell voltage. Compared to existing systems, our HCOONa production process demonstrated superior performance and efficiency. An experimentally grounded techno‐economic analysis (TEA) confirmed the excellent profitability of our HCOONa production process, offering a potential roadmap for achieving economic viability in CO_2_RR‐to‐formate applications.

## 2 Results and Discussion

### 2.1 Fabrication and Characterizations of GB‐Bi

**Figure 1:**
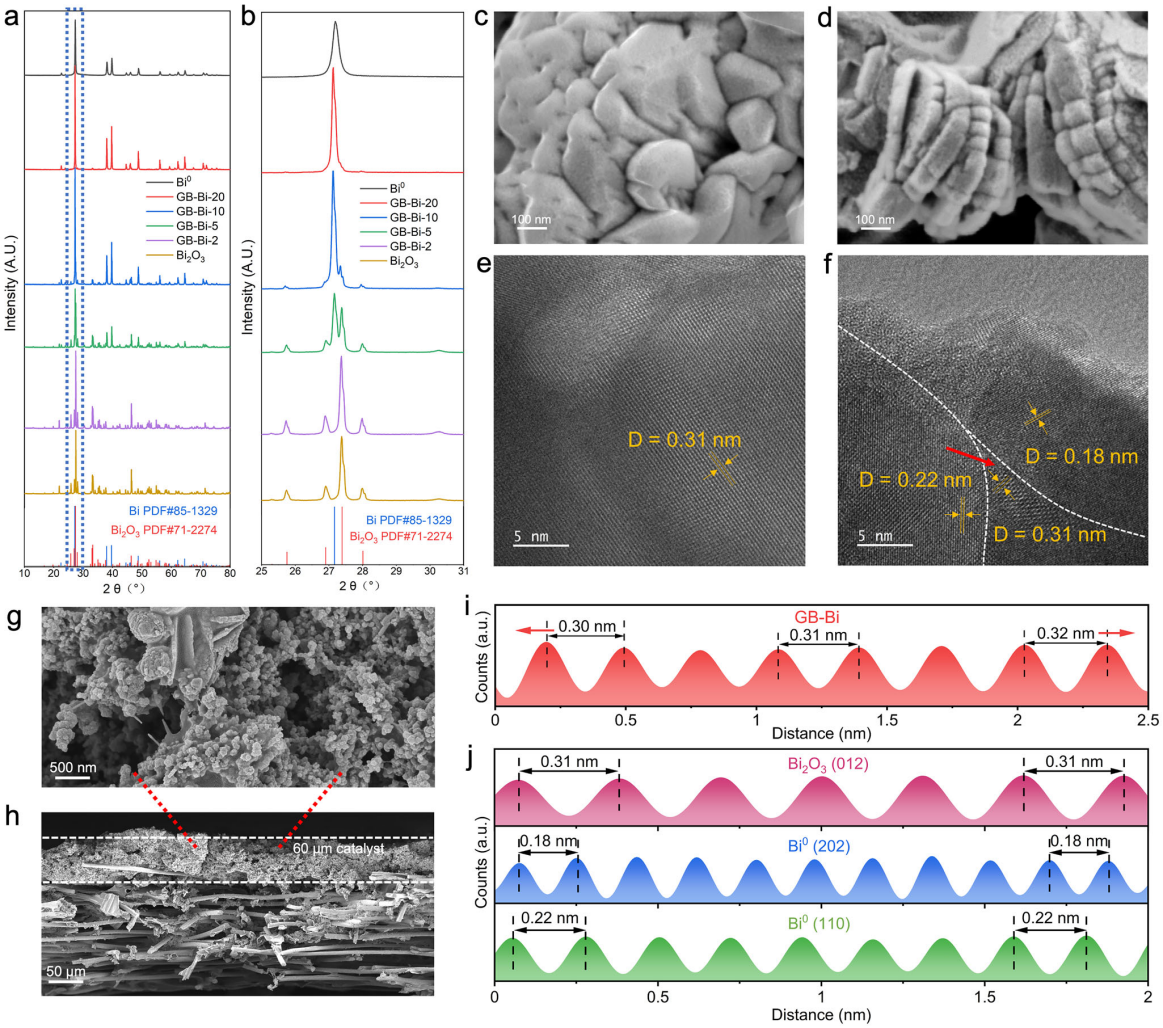
Characterization of Bi^0^, Bi_2_O_3_, and GB‐Bi. a) XRD patterns of Bi^0^, Bi_2_O_3_, and GB‐Bi‐x, with *x* indicating the quantity of reducing agent used (“Method”). b) Magnification of blue area in XRD patterns. c,d) SEM images of Bi_2_O_3_ (×100 000) and GB‐Bi (×100 000), respectively. e,f) HRTEM image of Bi_2_O_3_ and GB‐Bi, with lattice distance labeled, respectively. g) SEM images of GB‐Bi air‐brushed on gas diffusion layer (GDL) cathode (×20 000), h) Cross‐sectional view of the cathode (×200). i) Lattice stretching of Bi_2_O_3_ (012) in GB‐Bi from inverse fast Fourier transform of the direction of the arrow. j) Interplanar spacing measurements for Bi^0^ (110), Bi_2_O_3_ (012), and Bi^0^ (202).

Bi‐based catalyst is recognized as a promising catalyst for the CO_2_RR due to its high selectivity and activity in formate production.^[^ ^23^ ^]^ However, there is a necessity to enhance its current density and product selectivity.^[^ ^14^ ^]^ Research has demonstrated that an abundance of grain boundaries (GBs) provides an unsaturated coordination environment and many active sites,^[^ ^24^ ^]^ which has been demonstrated to improve the CO_2_RR performance across various catalysts.^[^ ^24^, ^25^ ^]^ Fan et al. reported a lithium (Li) tuning method to create abundant active GBs, yielding oxide‐derived Bi with small grains exhibiting distinct lattice orientation.^[^ ^20^ ^]^ However, the use of n‐butyl lithium (nBuLi) in this method is costly and the reaction process is complex, limiting its practical application. Previous research have demonstrated that the low‐energy surface Bi─O bonds of Bi_2_O_3_ can be readily attacked to create oxygen defects.^[^ ^26^ ^]^ Lei et al. used NaBH_4_ as a deoxidizer to achieve the synthesis of Bi_2_O_3_ with oxygen vacancy.^[^ ^26a^ ^]^ Learn from this idea, we introduce a straightforward chemical reduction process to generate abundant GBs by reducing Bi_2_O_3_ precursor with KBH_4_, which strips oxygen from the Bi_2_O_3_ lattice to form GB‐enriched Bi^0^‐BiO_x_. X‐ray diffraction (XRD) was utilized to compare the morphological differences of the Bi catalysts before and after KBH_4_ treatment. As depicted in **Figures** **1**a,b and S1 (Supporting Information), the Bi_2_O_3_ precursor gradually transforms into metallic Bi with increasing amounts of the reducing agent. X‐ray photoelectron spectroscopy (XPS) analysis further confirmed the formation of metallic Bi during the chemical reduction process (Figure S2, Supporting Information). Scanning electron microscopy (SEM) was employed to observe the morphological alterations in the Bi_2_O_3_ precursor before and after KBH_4_ treatment. Figure 1c and Figure S3 (Supporting Information) illustrate that the Bi_2_O_3_ precursor exhibited an aggregated nanoparticle morphology (≈100 nm). Post‐treatment with KBH_4_ resulted in a more dispersed and fragmented particle and the formation of layered stacks (Figure 1d), facilitating the exposure of additional active sites for CO_2_RR. The Brunauer–Emmett–Teller (BET) measurement has further confirmed that the surface area of GB‐Bi was 3 times higher than the Bi_2_O_3_ precursor (Figure S5, Supporting Information). Figure 1g,h demonstrate the SEM images of the surface and cross‐sectional views of the GB‐Bi‐Gas diffusion layer (GDL) cathode, respectively, showcasing the uniform application of the GB‐Bi catalyst onto the GDL, which ensures excellent catalytic performance and stability. The HR‐TEM image in Figure 1e reveals the uniform single crystalline nature of the Bi_2_O_3_ precursor (012). The interplanar distance variation map was calculated by the pixel intensity change with distance. As shown in Figure 1j, grains of Bi^0^ with distinct lattice orientation were observed in GB‐Bi pointed by the yellow arrow in Figure 1f, indicating the formation of diverse GBs.^[^ ^20^ ^]^ Figure 1i demonstrated that the interplanar distance stretched from 0.3 to 0.32 nm near the GB, indicating that the tensile strain of GB‐Bi is caused by grain boundaries.^[^ ^24a^ ^]^ The presence of GBs induces significant tensile stress and creates an unsaturated coordination environment, effectively optimizing the adsorption of intermediates and stabilizing the active site structure during the CO_2_RR process.^[^ ^24^ ^]^

### 2.2 Electrocatalytic CO_2_RR Performance of GB‐Bi Catalyst

**Figure 2:**
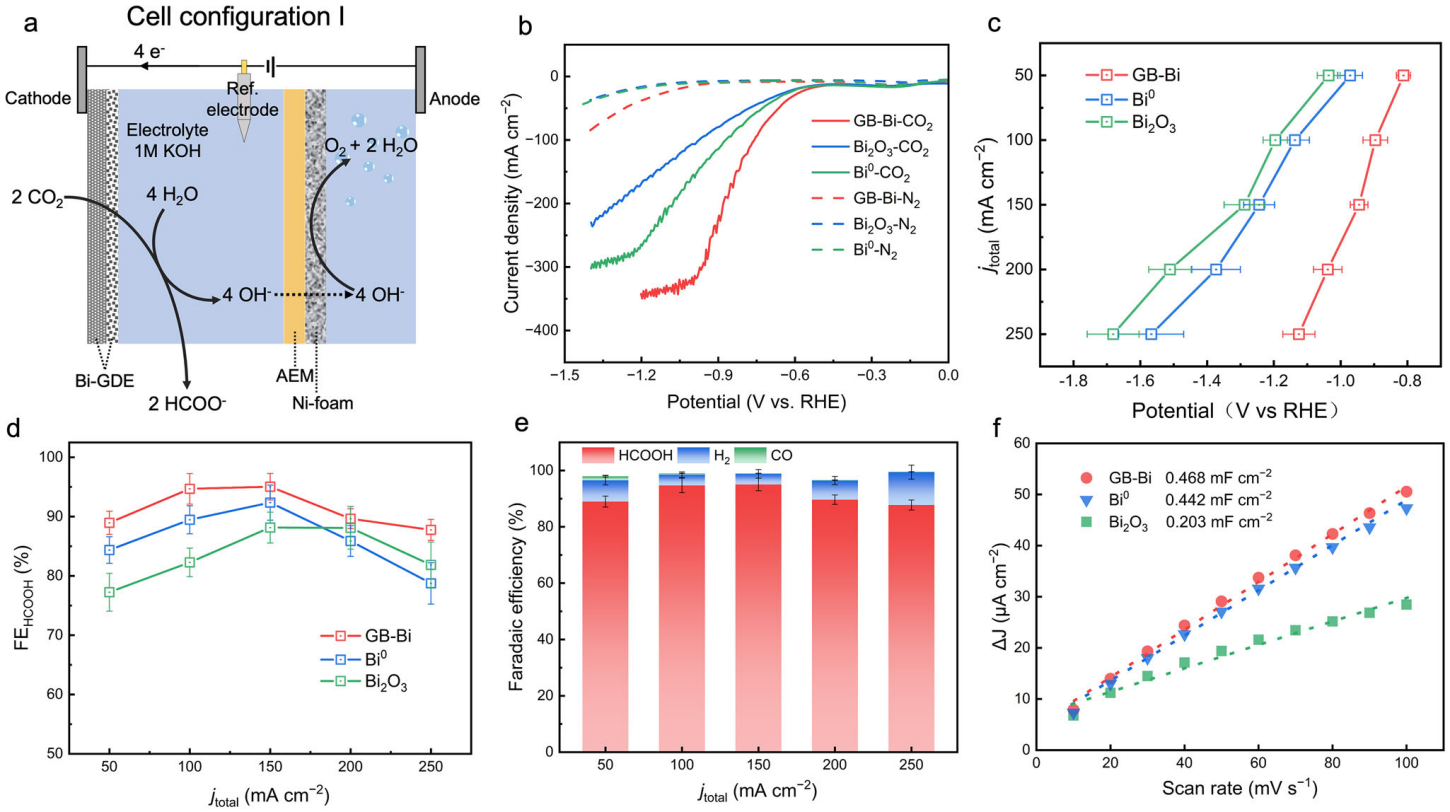
Design of Cell Configuration I (flow cell) and performance of Bi‐based catalyst. a) Schematic of Cell Configuration I and charge flow. b) Linear sweep voltammetry curves of GB‐Bi, Bi^0^, and Bi_2_O_3_ under N_2_ and CO_2_ atmospheres. c) Current density (*j*_total_) versus potential curves of GB‐Bi, Bi^0^, and Bi_2_O_3_ in Cell Configuration I using 1 m KOH electrolyte. d) Current density differences plotted against scan rates. e) The corresponding FEs of different products under different current densities. f) The comparison of formate FE among GB‐Bi, Bi^0^, and Bi_2_O_3_.

The performance of CO_2_RR‐to‐formate on GB‐Bi was first evaluated in Cell Configuration I (three‐electrode flow cell) with 1 m KOH solution being used as the electrolyte. As depicted in **Figure** **2**a, the Bi‐GDE electrode was prepared by air‐brushing a catalyst with a mass loading of 1 mg cm^−2^ onto a GDL. The anion exchange membrane (AEM) separated the anolyte and catholyte chambers (“Method”). Concurrently with the alkaline OER at the anode, OH^−^ generated by the cathodic CO_2_RR migrated through the AEM to the anode, thus maintaining the charge neutrality of the cell. Gas and liquid products were detected by gas chromatograph (GC) and nuclear magnetic resonance (NMR), respectively (“Methods”).

Among different Bi catalysts, the GB‐Bi demonstrated the lowest onset potential for CO_2_RR and showed an apparent increase in current density compared to Bi_2_O_3_ and Bi^0^ in linear sweep voltammetry curves (Figure 2b). At the potential of −0.96 V versus a reversible hydrogen electrode (RHE), a current density of 300 mA cm^−2^ was achieved. Compared with Bi_2_O_3_ and Bi^0^, GB‐Bi showed a lower overpotential across various current densities, and a difference of ≈0.5 V was observed at a current density of 250 mA cm^−2^ (Figure 2c). Consistently, GB‐Bi maintained high HCOO^−^ FE across various current densities (Figure 2d), achieving an FE of 95.1% at a current density of 150 mA cm^−2^. Compared with Bi_2_O_3_ and Bi^0^, GB‐Bi showed the greatest FEs of HCOO^−^ among various current densities (Figure 2d). The byproduct H_2_ was slightly increased at a current density of 50 and 250 mA cm^−2^ (Figure 2e). This trend may be attributed to the favorable kinetics of the HER at low overpotentials, coupling with limitations in CO_2_ mass transport at higher overpotentials.^[^ ^20^, ^27^ ^]^ But overall, GB‐Bi showed considerable activity and selectivity for HCOO^−^ production in CO_2_RR, emerging as the most effective catalyst in this experiment.

To unravel the electrochemical characteristics of different Bi catalysts, the electrochemical active surface area, and the electrochemical impedance spectroscopy (EIS) analysis was carried out. Compared with Bi_2_O_3_ (0.2 mF cm^−2^) and Bi^0^ (0.44 mF cm^−2^), GB‐Bi exhibited the greatest double layer capacitor (*C*_dl_) of 0.47 mF cm^−2^ (Figure 2f; Figure S6, Supporting Information). EIS measurements indicated that GB‐Bi had a lower equivalent charge transfer resistance (*R*_ct_) of 1.93 Ω compared with the Bi_2_O_3_ of 2.09 Ω and the Bi^0^ of 3.25 Ω (Figure S7, Supporting Information), illustrating the faster catalytic kinetics of GB‐Bi in CO_2_RR^[^ ^28^ ^]^ This result strongly indicated that GB‐Bi possesses both higher intrinsic activity and a larger active surface area compared to its counterpart.

To further validate the consistency of GB‐Bi under acidic conditions, a 0.5 m Na_2_SO_4_ solution at pH 2 was used as the electrolyte, with an IrO_2_‐Ti mesh as the acidic OER catalyst. As shown in Figure S8 (Supporting Information), GB‐Bi exhibited considerable performance under acidic conditions. The potential shifted from −0.88 to −1.29 V versus RHE as the current density increased from 50 to 200 mA cm^−2^. Similarly, GB‐Bi maintained high HCOO^−^ FEs (exceeding 81%) across various current densities under acidic conditions, achieving an FE of 88.1% at a current density of 200 mA cm^−2^. Compared to using 1 m KOH as the electrolyte, acidic conditions resulted in slightly higher overpotentials (≈−0.15 V) and slightly lower FEs (≈7% of HCOOH) across various current densities. This is attributed to the enhanced HER under acidic conditions,^[^ ^29^ ^]^ which competes with the CO_2_RR. Despite this, GB‐Bi still demonstrated substantial performance under acidic conditions.

### 2.3 Production of Electrolyte‐Free HCOOH Solution

**Figure 3:**
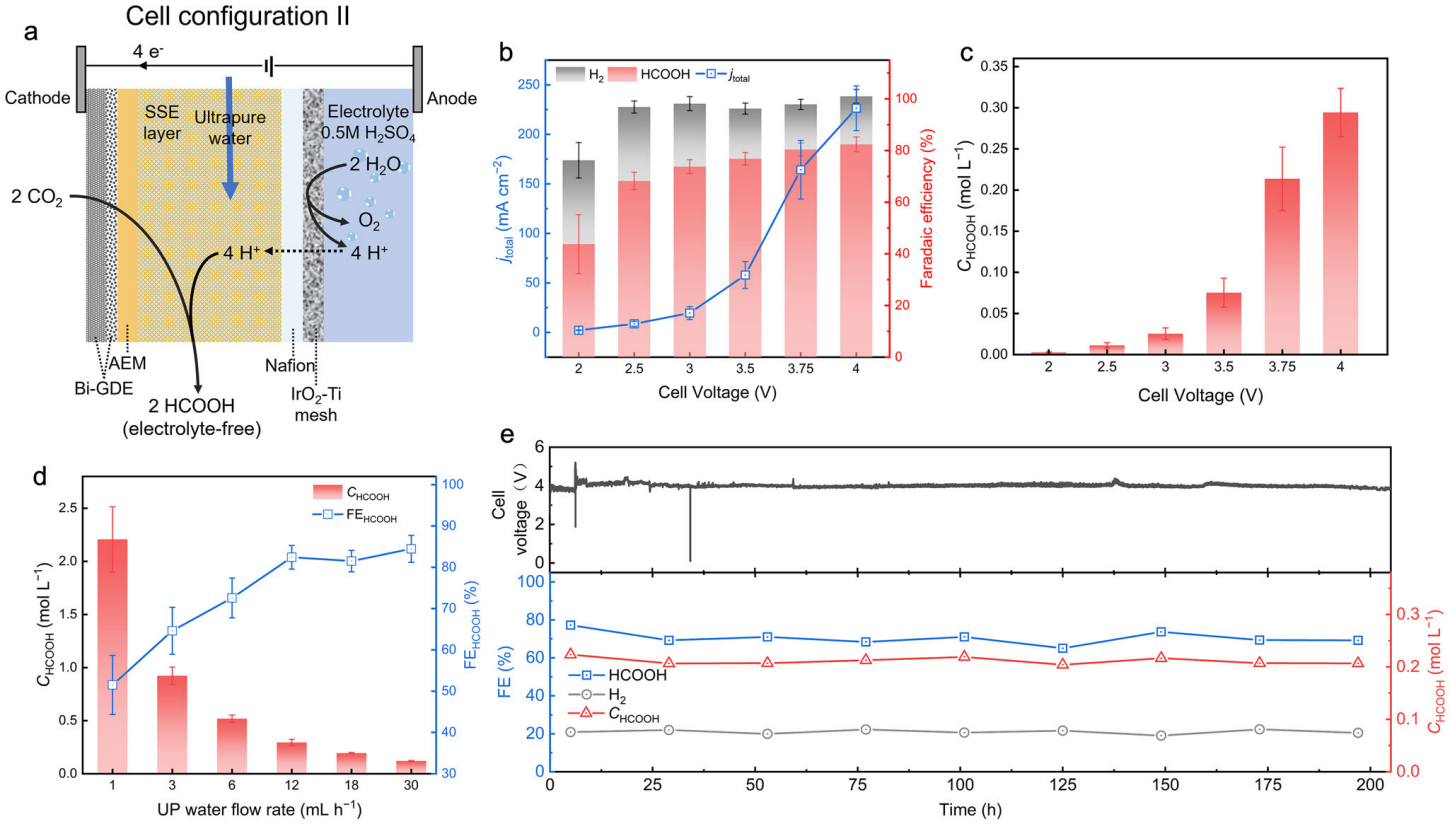
Design and performance of Cell Configuration II (MEA‐SSE cell) under GB‐Bi catalyst for continuous production of electrolyte‐free HCOOH solution. a) Schematic of Cell Configuration II and charge flow. b) The *j*_total_–*V* curves and corresponding FEs of different cell voltages. c) The dependence of HCOOH concentration on the cell voltage under a constant ultrapure water flow rate of 12 mL h^−1^. d) The dependence of HCOOH concentration on the ultrapure water flow rate under a constant cell voltage of 4 V, indicating that electrolyte‐free HCOOH solution (up to 2.2 m) can be continuously produced. e) Long‐term operation test of CO_2_ reduction to electrolyte‐free HCOOH solution at 200 mA cm^−2^. The FE of HCOOH is maintained at ≈70% over the 200 h continuous operation, up to 0.22 m electrolyte‐free HCOOH solution was continuously produced.

Extensive research has corroborated the advantages of employing solid‐state‐electrolyte (SSE) reactors for the production of the electrolyte‐free formic acid solution, which has been highlighted for its potential to significantly streamline the separation process and diminish the purification costs associated with formic acid production.^[^ ^4^, ^14^, ^16^, ^18^, ^19^, ^20^, ^30^ ^]^ Building on the demonstrated high activity of GB‐Bi in CO_2_RR‐to‐formate, we proceeded to evaluate its performance in cell Configuration II (two‐electrode SSE cell), as illustrated in **Figure** **3**a, which was specifically designed for the production of electrolyte‐free formic acid solution. A 0.5 m H_2_SO_4_ solution was employed as the anolyte. On the cathode side, HCOO^−^ generated by CO_2_RR migrated through the AEM toward the middle layer of SSE via the electric field. Concurrently, the protons H^+^ generated by acidic OER on the anode side migrated through the Nafion CEM toward the SSE layer, thus coupling HCOO^−^ to form HCOOH and maintaining the charge neutrality of the cell.^[^ ^19^ ^]^ The HCOOH product was released through ultrapure (UP) water instead of catholyte. IrO_2_‐Ti mesh, recognized for its stability and activity as an acidic OER catalyst,^[^ ^31^ ^]^ was equipped on the anode side. Sulfonated styrene‐divinylbenzene copolymer microspheres were employed as the SSE, which enables efficient cation transportation at room temperature between the anode and cathode (“Method”). The chemical equations of the reactions are presented below.

Cathode reaction:

(1) $$2CO_{2}+4e^{-}+4H^{+}\to 2HCOOH$$

Anode reaction:

(2) $$2H_{2}O-4e^{-}\to O_{2}+4H^{+}$$

Overall reaction:

(3) $$2CO_{2}+2H_{2}O\;\to 2HCOOH+O_{2}$$

As shown in Figure 3b, as the cell voltage was elevated from 2 to 3.5 V, the current density was increased to 57.9 mA cm^−2^, concurrent with a rise in HCOOH FE from 43.8% to 76.8%. Further increased the cell voltage to 4 V resulted in a rapid ascent in current density to 226.4 mA cm^−2^, while maintaining the HCOOH FE at ≈80%. Simultaneously, the concentration of the produced HCOOH was increased from 0.003 to 0.29 mol L^−1^ as the cell voltage increased from 2 to 4 V (Figure 3c). Throughout this process (Figure 3b,c), the flow rate of UP water was maintained at 12 mL h^−1^, a rate sufficient to prevent product accumulation and ensure an accurate assessment of intrinsic selectivity. These results suggest that GB‐Bi is well‐suited for operation in the SSE cell, facilitating the production of electrolyte‐free HCOOH solution. It's worth noting that the concentration of HCOOH in the solutions was effectively controlled by adjusting the flow rate of UP water. Under a cell voltage of 4 V, the HCOOH concentration was incrementally increased to a maximum of 2.21 m by slowing down the UP water flow rate to 1 mL h^−1^ (Figure 3d). However, the FE of HCOOH decreased from 81.5% to 51.5% as the UP water flow rate reduced from 12 to 1 mL h^−1^. This decrease may be attributed to the diffusion and crossover of the concentrated HCOOH to the anode, where it was subsequently oxidized prior to being released by the UP water flow.^[^ ^20^ ^]^ Subsequently, we further demonstrate the long‐term stability of cell Configuration II for the production of electrolyte‐free HCOOH solution, with a fixed current density of 200 mA cm^−2^ and a UP water flow rate of 12 mL h^−1^. During the continuous operation of 200 hours, stable production of ≈0.22 m HCOOH solution was achieved, with both cell voltage (≈3.8 V) and HCOOH FE (≈70%) remaining stable throughout the extended runtime (Figure 3e). Furthermore, the collected products were analyzed for possible impurities (such as bismuth, sodium, and potassium) using inductively coupled plasma atomic emission spectroscopy (ICP‐OES), confirming the exceptional purity of produced HCOOH solutions (Table S1, Supporting Information). Overall, GB‐Bi demonstrated promising CO_2_RR‐to‐HCOOH performance in the SSE cell, along with sustained long‐term stability, enabling the efficient production of electrolyte‐free HCOOH solutions.

### 2.4 Production of Electrolyte‐Free HCOONa Solution

**Figure 4:**
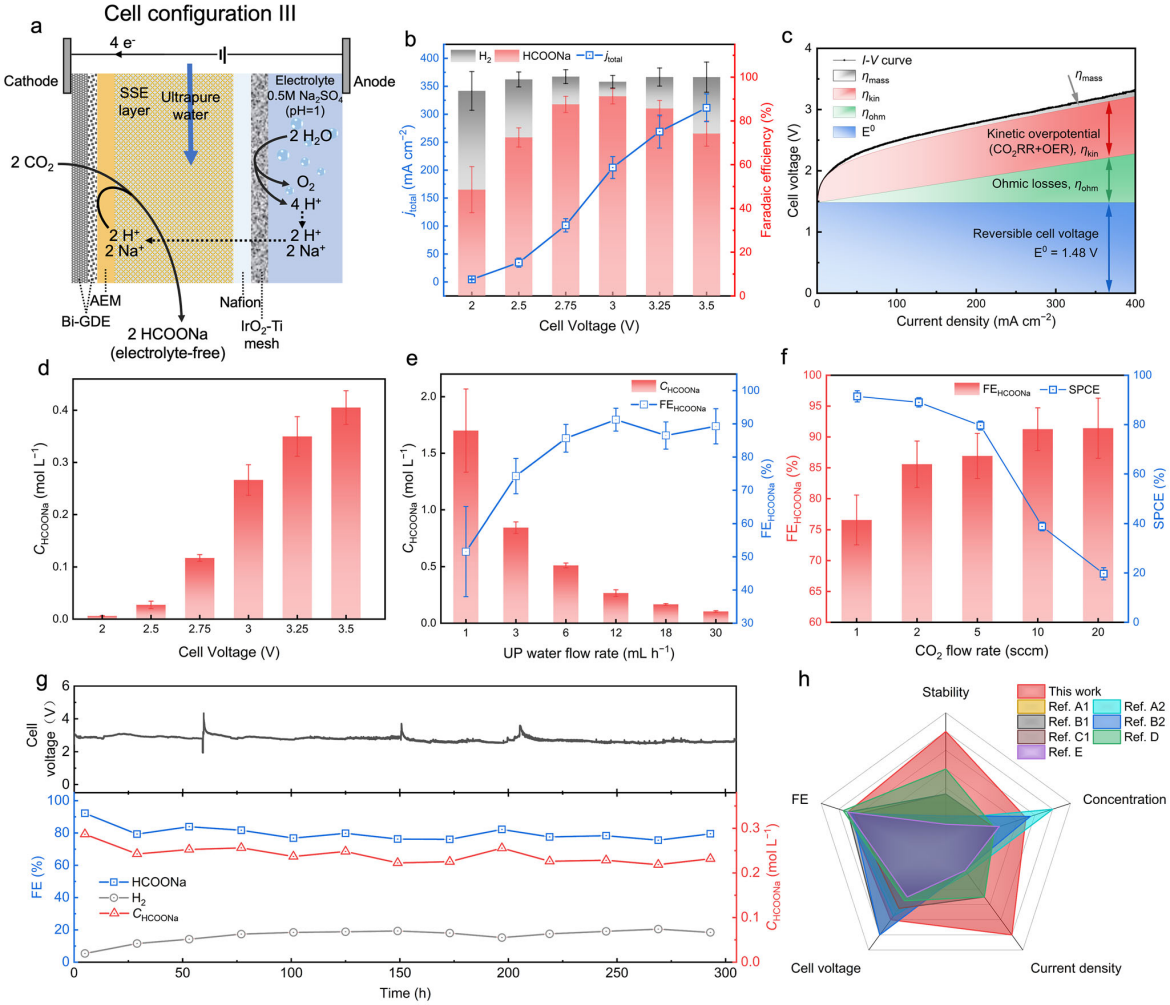
Design and performance of Cell Configuration III (MEA‐SSE cell) under GB‐Bi catalyst for continuous production of electrolyte‐free HCOONa solution. a) Schematic of Cell Configuration III and charge flow. b) The *j*_total_–*V* curves and corresponding FEs of different cell voltages. c) Individual contributions to cell voltage in Cell Configuration III, including reversible cell voltage, ohmic losses, kinetic overpotential, and mass transport overpotential. d) The dependence of HCOONa concentration on the cell voltage under a constant ultrapure water flow rate of 12 mL h^−1^. e) The dependence of HCOONa concentration on the ultrapure water flow rate under a constant cell voltage of 3 V, indicating that electrolyte‐free HCOONa solution (up to 1.7 m) can be continuously produced. f) Corresponding FEs and a single pass of carbon efficiency (SPCE) at different CO_2_ flow rates. g) Long‐term operation test of CO_2_ reduction to electrolyte‐free HCOONa solution at 200 mA cm^−2^. The FE of HCOONa was maintained at ≈80% over the 300 h continuous operation, and up to 0.27 m electrolyte‐free HCOONa solution was continuously produced. h) Radar chart summary for comparison of this work and other state‐of‐the‐art research on CO_2_ electrocatalytic production of electrolyte‐free HCOOH/HCOONa products.

Despite the successful production of electrolyte‐free HCOOH solutions in the SSE cell, the absence of alkali cations in the cathode limited the CO_2_RR performance, necessitating operation at a relatively high cell voltage of 3.8 V to overcome the high reaction overpotential, which in turn reduces energy efficiency (EE). Excessive electricity consumption not only significantly increases operational costs but also poses a risk of generating considerable Joule heat, potentially destabilizing the cell.^[^ ^16^ ^]^ To address this issue and broaden the application scope of the SSE cell, we proposed the cell Configuration III, as illustrated in **Figure** **4**a, which was specifically designed for the production of electrolyte‐free sodium formate (HCOONa) solution. The anolyte was replaced with 0.5 m Na_2_SO_4_ at pH 1.0, the protons H^+^ generated from acidic OER and the prevalent Na^+^ ions on the anode side migrated across the Nafion CEM toward the SSE layer, facilitating the production of HCOONa while preserving the charge neutrality of the cell. The HCOONa product was released through UP water instead of catholyte. The remaining setup parameters were consistent with those of Cell Configuration II (“Method”). The chemical equations of the reactions are presented below.

Cathode reaction:

(4) $$2CO_{2}+4e^{-}+2H^{+}+2Na^{+}\to 2HCOONa$$

Anode reaction:

(5) $$2H_{2}O\;-4e^{-}\to O_{2}+4H^{+}$$

Overall reaction:

(6) $$2CO_{2}+2H_{2}O\;+2Na^{+}\to 2HCOONa+2H^{+}+O_{2}$$

Figure 4b illustrates that elevating the cell voltage from 2 to 3 V resulted in a current density increase to 204.7 mA cm^−2^, concurrent with an increase in HCOOH FE from 48.6% to 91.3%. Upon further increased the cell voltage to 3.5 V, the current density increased further to 311.6 mA cm^2^, yet the HCOOH FE exhibited a decrease to 74.2%. This decrease in FE at higher voltages and higher current densities may be attributed to the concurrent generation of a higher concentration of hydroxide ions (OH^−^) during CO_2_RR, which increased the CO_2_ permeation flux and consequently accelerated the decay of CO_2_RR performance.^[^ ^20^ ^]^ In comparison to Cell Configuration II, the HCOONa production process in Cell Configuration III exhibited significantly higher current densities among various cell voltages and a maximum HCOO^−^ FE of 91.3%. At the same current density (100–200 mA cm^−2^), a cell voltage reduction of ≈1000 mV was observed in the HCOONa production process (Figure S9, Supporting Information). A detailed analysis of contributions to the cell voltage in cell configurations II and III was conducted. As shown in Figure 4c and Figure S10 (Supporting Information), in cell configuration III, the main reason for the decrease in cell voltage was the reduction of kinetic overpotential, followed by the reduction of ohmic losses and the decrease in mass transport overpotential. This result confirmed that the involvement of Na^+^ ions enhanced the CO_2_RR performance as expected. Simultaneously, the concentration of the HCOOH production was increased from 0.01 to 0.41 mol L^−1^ as the cell voltage increased from 2 to 3.5 V. Throughout this process (Figure 4c,d), the flow rate of UP water was still maintained at 12 mL h^−1^. Similarly, the concentration of HCOONa in the solutions was effectively controlled by adjusting the flow rate UP water (Figure 4e) and followed a similar trend to the HCOOH production process shown in Figure 3d. A maximum HCOONa concentration of 1.7 m was achieved at a UP water flow rate of 1 mL h^−1^, corresponding to an HCOONa FE of 48.6%. It has been evidenced that significant carbon loss exceeding 50% could be caused by carbonate precipitation in alkaline electrolytes,^[^ ^4^, ^17^ ^]^ leading to markedly low single pass of carbon efficiency (SPCE). Conversely, our HCOONa production process achieved an impressive SPCE of >90% by reducing the inlet CO_2_ flow rate to 1 sccm, with a slightly reduced FE to 76% due to the increased HER kinetics (Figure 4f). Subsequently, we further examined the long‐term stability of cell Configuration III in the production of electrolyte‐free HCOONa solutions, operating at a fixed current density of 200 mA cm^−2^ and a UP water flow rate of 12 mL h^−1^. Compared to the HCOOH production process (Figure 4g), Cell Configuration III for HCOONa production exhibited even longer stability (300 h), higher HCOONa FE (≈80%), and lower cell voltage (≈2.8 V), culminating in a continuous and stable production of ≈0.27 m HCOONa solution, with both cell voltage and HCOOH FE remaining stable over the extended runtime. The long‐stability in this work could be attributed to the following reasons: 1) The continuous‐flow operation reduced the residence time of alkali cations. 2) The MEA‐SSE cell allowed alkali cations to migrate only through electric field and diffusion effects, and the presence of AEM further reduced the likelihood of alkali cations migrating into the GDL.^[^ ^19^ ^]^ 3) PTFE‐treated GDL (“Method”) enhanced hydrophobicity, reduced the probability of flooding and salt precipitation, and significantly improved stability.^[^ ^4b^ ^]^ ICP‐OES, IC, and NMR were used to confirm the exceptional purity of produced HCOONa solutions (Table S1, Figures S11,S12, Supporting Information). In comparison to HCOONa products mixed with electrolytes, such as 1 m KOH or KHCO_3_, the electrolyte‐free HCOONa product can be concentrated via a straightforward distillation and filtration process. To validate this approach, 240 mL of electrolyte‐free HCOONa solution collected from cell Configuration III at 200 mA cm^−2^ was concentrated, resulting in a white crystalline precipitate weighing 3.98 g, and XRD pattern further confirmed the exceptional purity of the produced HCOONa precipitate (Figure S12, Supporting Information). This demonstrates that the separation of electrolyte‐free sodium formate (HCOONa) products is both straightforward and cost‐effective. Compared to the existing state‐of‐the‐art research on the production of electrolyte‐free HCOO^−^ products in SSE cells,^[^ ^14^, ^19^, ^20^, ^30^, ^32^ ^]^ our strategy for the HCOONa production process demonstrates exceptional performance and stability (Figure 4h, Table S2, Supporting Information). Additionally, K^+^ ions exhibited a similar promotional effect alongside Na^+^ ions (Figure S13, Supporting Information), indicating MEA‐SSE cell was well‐suited for the production of other formate salts, such as potassium formate (Figure S14, Supporting Information). To meet the demands for industrial applications, a scaled‐up MEA‐SSE electrolyzer with an effective area of 4 cm^2^ was employed. A 0.5 m Na_2_SO_4_ at pH 1 was employed as the anolyte. As shown in Figure S15 (Supporting Information), the cell voltage increased from 2.02 to 4.54 V as the total current increased from 0.05 to 2 A. The FEs of HCOONa increased from 45.3% to 89.3% as the total current increased from 0.05 to 1 A. Further increasing the total current to 2 A led to a decrease in the FE of HCOONa to 64.5%. Overall, the scaled‐up electrolyzer with a high total current demonstrates that the HCOONa production process holds high potential for industrial applications. These advancements expand the application range of SSE cells and potentially facilitate the industrialization of CO_2_RR‐to‐formate conversion processes.

### 2.5 Post CO_2_RR Characterization

**Figure 5:**
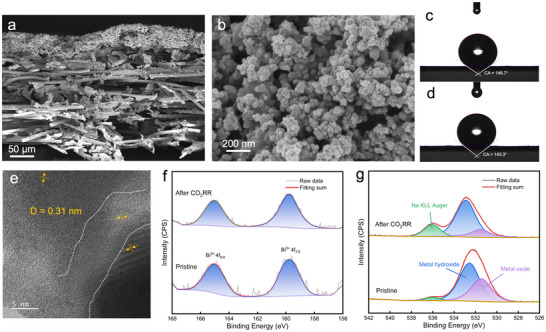
SEM images of a) Cross‐sectional view (×200) and b) surface view (×50 000) of the cathode after CO_2_RR. c,d) Contact measurements on the cathode before and after electrolysis. e) The HRTEM image of GB‐Bi, with the lattice distances labeled, respectively. f) O1s XPS spectra of pristine cathode and after electrolysis. g) Bi4f XPS spectra of pristine cathode and after electrolysis.

To assess the catalyst stability in CO_2_RR, we characterized the GB‐Bi‐GDL cathode after a 20 h reaction. The cathode maintained stability of morphology and no agglomeration tendency was observed (**Figure** **5**a,b). STEM‐EDS mapping revealed a uniform distribution of Bi, C, and O across the catalyst (Figure S16, Supporting Information). HR‐TEM (Figure 5e) reveals that the GB‐Bi structure remains GBs‐enriched after the reaction. On the other hand, the cathode maintained consistent hydrophobicity during CO_2_RR (Figure 5c,d), indicating the retention of the stable three‐phase interface, which was a key factor for the exceptional durability in CO_2_RR.^[^ ^4^, ^33^ ^]^ XRD spectra (Figure S17, Supporting Information) show peaks corresponding to Bi_2_O_3_, overlapping with signals from the carbon‐based GDL, and the signal of Bi_2_O_3_ did not weaken after the reaction. High‐resolution Bi 4f and O1s XPS spectra further confirmed the stable presence of Bi^3+^ before and after CO_2_RR (Figure 5f,g). After CO_2_RR, significant increases in metal hydroxide and Na KLL Auger signals suggest surface interaction of GB‐Bi and Na^+^. Consequently, GB‐Bi demonstrates superior performance and stability in CO_2_RR, positioning it as a promising electrocatalyst for HCOO^−^ production.

### 2.6 CO_2_RR Mechanistic Study

**Figure 6:**
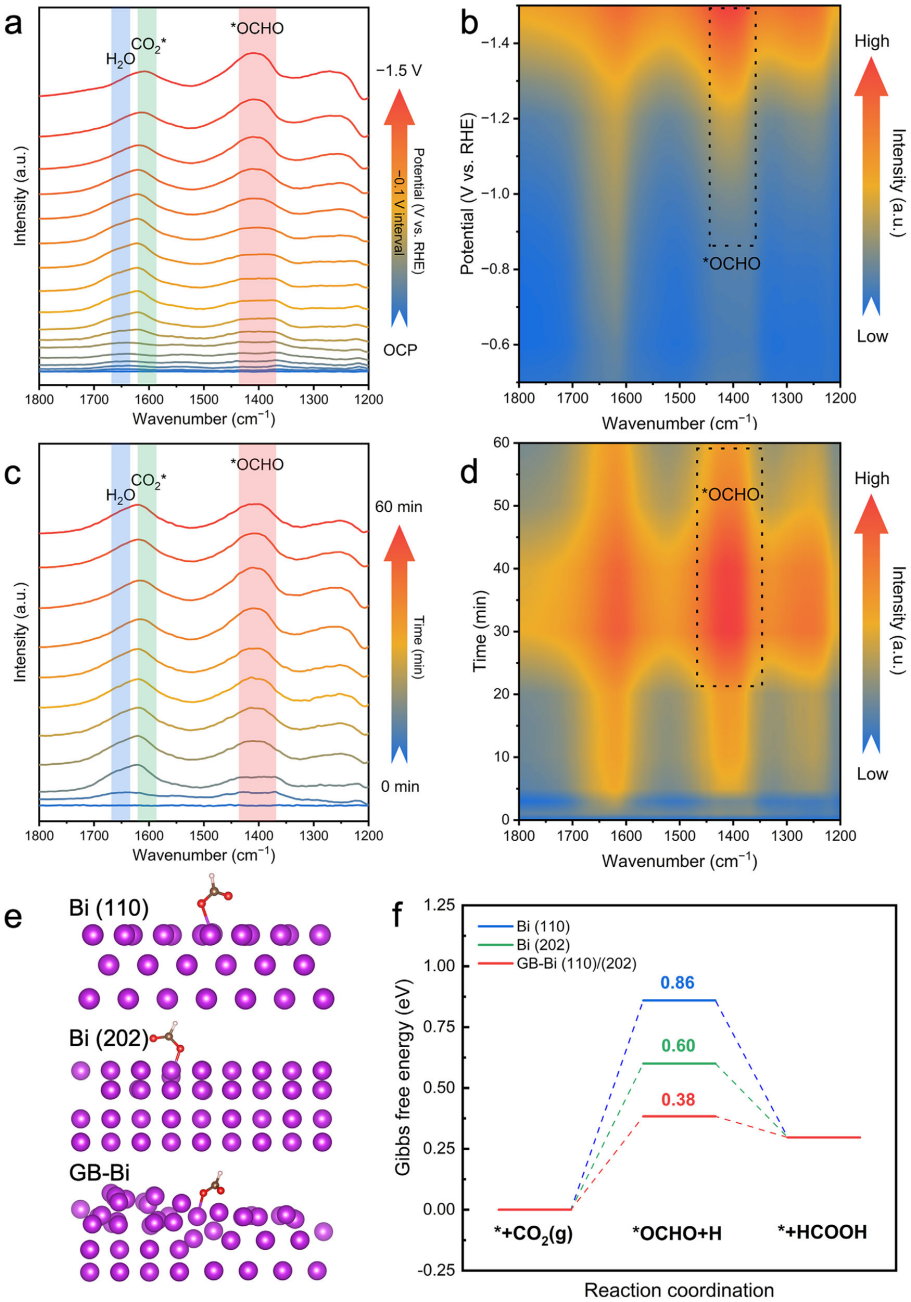
a) In situ ATR‐FTIR spectra of GB‐Bi under various potentials (vs RHE). b) Corresponding contour map. c) Time‐dependent In situ ATR‐FTIR spectra of GB‐Bi at −1.2 V versus RHE. d) Corresponding contour map. 0.5 m Na_2_SO_4_ solution at pH = 2 was used as electrolyte. e) Side‐views of the adsorption geometries of the intermediates *OCHO on Bi (110), Bi(202) and GB‐Bi(110)/(202) surface. f) Calculated free‐energy diagrams for CO_2_RR over Bi (110), Bi(202) and GB‐Bi(110)/(202) surface at pH = 2.

To illuminate the mechanism of the CO_2_RR process, in situ, attenuated total reflectance Fourier transform infrared (ATR‐FTIR) spectroscopy was employed to monitor reaction intermediates (**Figure** **6**a–d; Figure S18, Supporting Information). As depicted in Figure 6a,b, applying potentials ranging from −0.1 to −1.5 V versus RHE to the GB‐Bi catalyst induced vibrational peaks near 1400 cm^−1^, which intensified with increasing negative potential. This peak is attributed to the key intermediate *OCHO in HCOO^−^ formation.^[^ ^34^ ^]^ The infrared signal at 1600 cm^−1^, corresponding to adsorbed CO_2_*, indicates the activation of CO_2_ molecules on the catalyst surface, with greater intensity at more negative potentials, suggesting higher CO_2_ activation.^[^ ^35^ ^]^ Concurrently, the infrared signal at 1650 cm^−1^, corresponding to adsorbed H_2_O, gradually decreases as the potential becomes more negative, indicating the continuous consumption of H_2_O during the CO_2_RR process, which serves as a proton donor.^[^ ^34a^ ^]^ Time‐dependent spectra further corroborate these observations, with a distinct *OCHO vibrational signal emerging after 5 min of reaction at −1.2 V versus RHE (Figure 6c,d).

To further elucidate the mechanisms underlying the enhanced activity for HCOOH formation, density functional theory (DFT) was conducted. Three Bi catalyst models of pristine Bi (110), pristine Bi (202), and GB‐Bi (012)/(202) with grain boundaries were constructed, for investigating HCOOH formation in CO_2_RR. The Bi(110) and Bi(202) surfaces were selected to present flat Bi in alignment with experimental results. The *OCHO intermediate, consistent with in situ ATR‐FTIR findings, was considered crucial for HCOOH formation on Bi catalysts.^[^ ^34a^ ^]^ Therefore, calculations focused on the *OCHO pathway. Figure 6e and Figure S19 (Supporting Information) display the side‐views and top‐views of the adsorption geometries of the *OCHO intermediate on Bi(110), Bi(202), and GB‐Bi surfaces. The corresponding free energy diagrams (Figure 6f) reveal that *OCHO formation was the rate‐limiting step for HCOOH formation on all three surfaces, with calculated barriers of 0.86 eV for Bi(110), 0.60 eV for Bi(202), and 0.38 eV for GB‐Bi. These results demonstrate that the introduction of grain boundaries significantly reduces the free energy barrier for HCOOH formation.

### 2.7 Techno‐Economic Analysis

**Figure 7:**
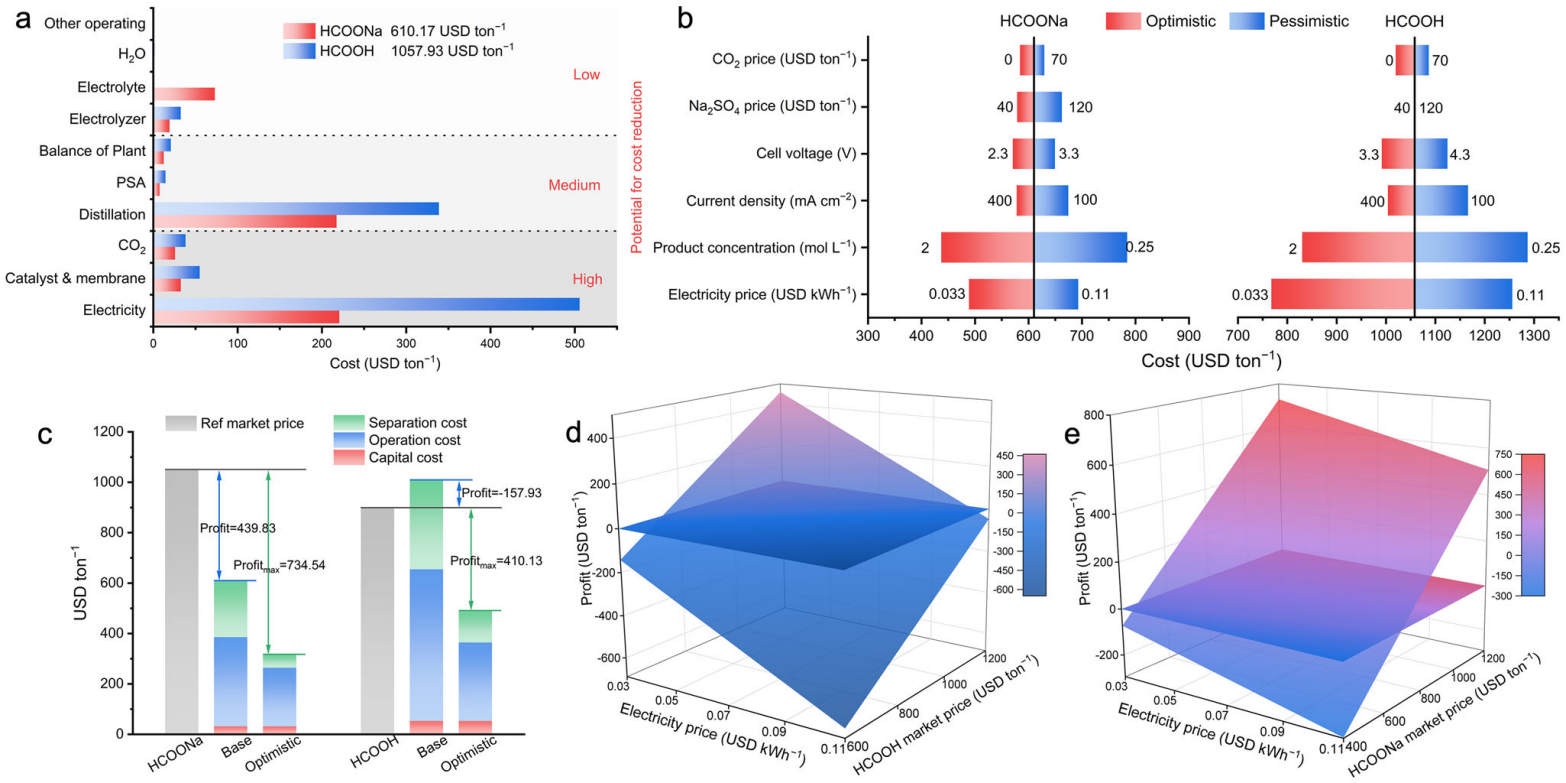
a) The subdivided cost of the entire process for HCOOH and HCOONa productions. b) Sensitivity analysis for HCOOH and HCOONa productions. c) The total cost and profit for HCOONa and HCOOH productions based on the optimized operational conditions (base) and projected improvements in cell performance and power costs (optimistic) conditions. The profitability for d) HCOOH and e) HCOONa productions under the fluctuation of the market.

In the context of practical applications, economic viability is paramount in assessing the feasibility of implementing CO_2_RR technologies. Herein, we conducted a techno‐economic analysis (TEA) to evaluate the cost associated with producing electrolyte‐free HCOONa and HCOOH solutions under the optimized operational conditions mentioned above, based on the performance of GB‐Bi in MEA‐SSE cells (Table S3, Supporting Information). First, The TEA framework including CO_2_ electrolysis and subsequent product separation was established. As shown in Figure S20 (Supporting Information), a distillation process was employed to concentrate the solutions. Nonreactive CO_2_ and gaseous products were separated by a pressure‐swing adsorption (PSA) system, with nonreactive CO_2_ being recycled back into the electrolysis process. As shown in **Figure** **7**a, our analysis revealed that electricity was identified as the predominant cost component, accounting for 36.2% (220.68 USD ton^−1^) and 47.8% (505.73 USD ton^−1^) of the total production costs for HCOONa and HCOOH production, respectively. Distillation costs followed but were significantly lower compared to conventional liquid electrolyte‐based CO_2_RR processes, in which liquid separation costs exceed 80% of total costs.^[^ ^16^ ^]^ Given that HCOONa produced via MEA‐SSE cells exhibits exceptional purity akin to that of HCOOH products in MEA‐SSE cells, their purification processes and associated unit costs are comparable. Electrolyte‐free HCOONa and HCOOH production processes exhibited markedly reduced liquid separation costs, accounting for 35.6% (217.39 USD ton^−1^) and 36.8% (388.81 USD ton^−1^) of total costs, respectively. We identified that the cost of electricity, catalysts and membranes, and CO_2_ can be potentially reduced through strategies such as utilizing onshore wind power, developing efficient catalysts and membranes, and accessing CO_2_ tax credits.^[^ ^2^, ^36^ ^]^ It must be noted that while the HCOONa production process incurs additional costs for Na_2_SO_4_ input (73.08 USD ton^−1^, 12.0% in the HCOONa production process), the reduction in cell voltage and enhancement in FEs yields substantial cost savings, particularly in electricity costs (285.05 USD ton^−1^ in electricity). The key parameters that significantly influence the production costs of each product were performed on a single‐variable sensitivity analysis. As shown in Figure 6b, among all parameters, electricity prices were found to have the most significant impact on HCOOH production cost and the second potential for cost reduction in HCOONa production. Switching to onshore wind power as the driving force (0.033 USD kWh^−1^)^[^ ^37^ ^]^ instead of industrial electricity (0.08 USD kWh^−1^),^[^ ^37a^ ^]^ the costs of HCOOH and HCOONa production could significantly reduce by 290.26 and 121.37 USD ton^−1^, respectively. Optimizing the concentration of electrolyte‐free products would be the next priority. In detail, 173.34 and 227.8 USD ton^−1^ in cost reduction for HCOONa and HCOOH production, respectively, are expected when the product concentration reaches 2 m. The current density of 200 mA cm^−2^ was deemed as a suitable parameter because, the impact of current density on cost benefits is nonlinear. While doubling it to 400 mA cm^−2^ offers limited additional cost benefits, reducing the current density to half (100 mA cm^−2^) results in a greater cost increase than doubling it. In the base case condition, considering the market price, the HCOONa production process is profitable of 439.83 USD ton^−1^, while the HCOOH production process incurs a loss of 157.93 USD ton^−1^ (Figure 6c). Optimistic estimates based on sensitivity analysis suggest maximum profits of 734.54 USD ton^−1^ for HCOONa and 410.13 USD ton^−1^ for HCOOH, respectively. In addition, based on the above analysis, we also analyzed the electricity price fluctuation within the range of 0.033–0.11 USD kWh^−1^ and commercial HCOOH and HCOONa product price ranges of 400–1200 USD ton^−1^ (Figure 6d,e). These analyses underscore the excellent potential for achieving economic viability in the electrolyte‐free HCOONa production process. Given the system's capacity to produce a variety of products simply by adjusting the anolyte composition without altering the fundamental structure of the cell, it possesses the flexibility to switch production of HCOOH and HCOONa. This adaptability enables the system to effectively accommodate market demands and mitigate the impact of price fluctuations. Therefore, the experimentally grounded TEA presents a potential roadmap for achieving economic viability in CO_2_RR‐to‐formate.

## 3 Conclusion

In summary, we have successfully developed a novel approach for the tunable production of both electrolyte‐free HCOOH solution and electrolyte‐free HCOONa solution via CO_2_RR in an MEA‐SSE cell. A grain boundary enriched GB‐Bi catalyst was employed for CO_2_RR, achieving HCOO^−^ FEs of ≈90% across a broad current density ranging from 50 to 250 mA cm^−2^ at low potentials between −0.85 to −1.15 V versus RHE. The anolyte composition in the MEA‐SSE cell configuration was found to be a key factor in determining the product form. Employing a 0.5 m H_2_SO_4_ solution as the anolyte, ≈0.22 m electrolyte‐free HCOOH solution was continuously produced over 200 h. Switching the anolyte to 0.5 m Na_2_SO_4_ at pH 1 for HCOONa production resulted in enhanced stability with the continuous operation being extended to 300 h, and a reduction of cell voltage of ≈1000 mV, leading to the sustained generation of ≈0.27 m electrolyte‐free HCOONa solution. The obtained HCOONa solution exhibited exceptional purity and could be readily concentrated via a straightforward evaporation process. Compared to existing systems, our HCOONa production process demonstrated superior performance and stability. In situ ATR‐FTIR spectra and DFT calculations demonstrated that GB‐Bi has stronger binding energy to the key intermediate OCHO* compared to flat Bi, thereby enhancing HCOO^−^ production. Experimentally grounded TEA underscored the excellent profitability of our HCOONa production process. Future research can be focused on the production of high‐concentration products to further enhance the economic viability in CO_2_RR‐to‐formate applications.

## Conflict of Interest

The authors declare no conflict of interest.

## Supporting information



Supporting Information

## Data Availability

The data that support the findings of this study are available from the corresponding author upon reasonable request.
