# Supplementary material for: A Tunable Strategy for Continuous Production of Electrolyte‐Free Formic Acid and Sodium Formate in a Solid‐State‐Electrolyte Based Electrocatalytic CO2 Reduction System
Source: Adv Sci (Weinh). 2025 Jun 30;12(36):e08152. doi: 10.1002/advs.202508152 (PMC12462986; doi:10.1002/advs.202508152)
Supplement: Supplementary file 1 — Supporting Information [file ADVS-12-e08152-s001.docx]

**Supplementary Information**

**A tunable strategy for continuous production of electrolyte-free formic acid and sodium formate in a solid-state-electrolyte based electrocatalytic CO_2_ reduction system**

Jinrui Guo ^a^, Wenqiang Qi ^a^, Rongrong Mo ^a^, Feiyi Yuan ^a^, Lin Wang ^a, b^, Yongmei Li ^a, b, *^

a State Key Laboratory of Water Pollution Control and Green Resource Recycling, College of Environmental Science and Engineering, Tongji University, Shanghai, 200092, People's Republic of China

b Shanghai Institute of Pollution Control and Ecological Security, Shanghai, 200092, People's Republic of China

*Corresponding author: Yongmei Li; Email: liyongmei@tongji.edu.cn

No.1239 Siping Road, Yangpu District, Shanghai

**Method**

**Chemicals and Materials.** Sodium hydroxide (NaOH, 99%), potassium hydroxide (KOH, 99%), sodium sulfate (Na_2_SO_4_, 99%), potassium sulfate (K_2_SO_4_, 99%), bismuth nitrate pentahydrate (Bi(NO_3_)_3_•5H_2_O, 99.9%, potassium borohydride (KBH_4_, 99%), HPLC grade isopropanol (99.7%), sulfuric acid (H_2_SO_4_, about 98%), and dimethyl sulfoxide (DMSO, 99.9%) were purchased from Sinopharm Chemical Reagent Co., Ltd. D_2_O (99.9 atom % D) was purchased from Meryer (Shanghai) Biochemical Technology Co., Ltd. All chemicals were used without further purification. Nafion D520 dispersion (5 wt%) and Nafion N115 cation exchange membrane (CEM) were obtained from DuPont.  PTFE emulsion (60 wt%) was purchased from Aladdin Biochemical Technology Co., LTD. YLS-30T gas diffusion layer (GDL), FAA-PK-130 and Sustainion X37-50-grade-FA anion exchange membrane (AEM), Ni-foam and IrO_2_-Ti mesh were obtained from Suzhou Sinero Technology Co., LTD. High-purity nitrogen gas (N_2_, 99.999%) and carbon dioxide (CO_2_, 99.995%) were purchased from Wendong Chemical Products Co., LTD. The ultrapure water was obtained from milli-Q system.

**Synthesis of Bi_2_O_3_.** Typically, 2 g of Bi(NO3)_3_•5H_2_O were dissolved in 50 mLof ultrapure water while stirring at 600 rpm to prepare Solution A. Next, 0.5 g of NaOH was dissolved in 50 ml of ultrapure water to form Solution B. Subsequently, the Solution B was quickly added to the Solution A, and the mixture was stirred at 400 rpm for 30 min. The resulting white product was collected by centrifugation and washed with deionized water and alcohol. The obtained product was then transferred into a crucible and heated in a muffle furnace at a preset temperature of 500 °C for 60 min with a heating rate of 5 °C min^−1^. After the sintering process, the product was cool down at room temperature and was labeled as Bi_2_O_3_.

**Synthesis of GB-Bi.** GB-Bi was synthesized using a simplified chemical reduction procedure. Solution A was prepared by dispersing 2 g Bi_2_O_3_ in 50 ml ultrapure water under 600 rpm stirring to form a water suspension. Next, x/20 g KBH_4_ was dissolved in 50 ml ultrapure water to form Solution B (a x g L^−1^ KBH_4_ solution). Then, Solution B was slowly injected to Solution A over ~10 min, followed by a 30 min reaction until gas evolution ceased. Finally, the product was collected via filtration, washed three times with ultrapure water and alcohol, and then freeze-dried for 12 h. The obtained product was labeled as GB-Bi-x, with x being 5 in GB-Bi unless otherwise specified.

**Synthesis of Bi^0^.** Typically, 2 g Bi(NO_3_)_3_•5H_2_O was dissolved in 50 ml ultrapure water under 600 rpm stirring to form Solution A. Subsequently, 1 g KBH_4_ was dissolved in 50 ml ultrapure water to form Solution B. Then, Solution B was slowly injected to Solution A over ~10 min, followed by a 30 min reaction until gas evolution ceased. Finally, the product was collected by filtration, washed by ultrapure water and alcohol for 3 times, and then freeze-dried for 12 h. The obtained product was labeled as Bi^0^.

**Characterization of the catalysts.** X-ray diffraction (XRD) patterns were acquired using a Bruker D2 Phaser X-ray diffractometer equipped with Cu Kα radiation, with a 2θ range from 20° to 80°. Scanning electron microscopy (SEM) was performed on a ZEISS Sigma 300 field emission SEM operating at an acceleration voltage of 3 kV. Energy-dispersive X-ray spectroscopy (EDS) and mapping were conducted using an Oxford Xplore 30 at an acceleration voltage of 15 kV. High-resolution transmission electron microscopy (HR-TEM) was carried out with JEOL JEM-F200 (Japan) at an acceleration voltage of 200 kV. X-ray photoelectron spectroscopy (XPS) was conducted using a Thermo Scientific K-Alpha XPS system, employing monochromated Al Kα radiation at 1486.6 eV. All XPS data were calibrated by shifting the binding energy of the detected carbon C1s peak to 284.8 eV. Contact angle was performed using a JY-82C goniometer (Dingsheng, China), employing the sessile drop method to determine the contact angle. N_2_ adsorption-desorption analysis was performed using ASAP 2460 (Micromeritics Instrument Corporation, USA). The Brunauer-Emmett-Teller (BET) specific surface area and pore size distribution was determined at 77 K, with degassing at 393 K.

**Electrochemical CO_2_ reduction.** All the electrochemical measurements were performed using a CHI 760E electrochemical workstation (CH Instrument, Shanghai, China). In the flow cell configuration (cell configuration I), a GDL coated with Bi-based catalyst, Ni foam (1.5×1.5 cm, 1 mm thick) and Hg/HgO (1 M KOH) served as the cathode, anode, and reference electrode, respectively. To prepare the cathode, a YLS-30T GDL (1.5×1.5 cm) was first sprayed by 0.1 mL of 2 wt% PTFE emulsion and then heated in a muffle furnace at a preset temperature of 160 °C for 60 min with a heating rate of 5 °C min^−1^ to mitigate electrowetting during the electrocatalytic process. The electrocatalyst inks were formulated by mixing 10 mg of the catalysts, 0.4 ml ultrapure water, 0.5 ml isopropanol, 10 µl of 2 wt% PTFE emulsion and 0.1 mL of Nafion D520 dispersion, followed by ultrasonication for 30 min. The catalyst ink was then air-brushed onto a YLS-30T GDL (1.5×1.5 cm) with a catalyst mass loading of 2 mg cm^−2^. Two titanium flow chambers were installed on the cathode and anode sides. A 3 mm thick Polyetheretherketone (PEEK) sheet, featuring a 1.0×1.0 cm hole, was used to separate the cathode and anode compartments of the flow cell. A FAA-PK-130 AEM was sandwiched between the PEEK sheet and the anode titanium flow chamber to separate the cell chambers. This configuration enabled interaction between the catalyst layer and the flowing liquid electrolyte, and the geometric surface area of the catalyst was fixed at 1 cm^2^. Cathode titanium flow chamber was fed with 20 sccm CO_2_, while PEEK sheet and anode titanium flow chamber were supplied with 1.0 M KOH at a flow rate of 5 mL min^−1^. All potentials referenced against Hg/HgO were converted to the reversible hydrogen electrode (RHE) scale in this work, using the equation: E_RHE_ = E_Hg/HgO_ + 0.098 V + 0.0591 × pH. The linear sweep voltammetry (LSV) curves were recorded at a scan rate 10 mV s^−1^ under CO_2_ or Ar atmosphere from 0 to −1.3 V vs. RHE. The ECSA was calculated using the formula: ECSA = R_f_ × S, where S denotes the actual surface area of the smooth metal electrode, equivalent to the geometric area of the working electrode. The roughness factor (R_f_) was determined using the formula: Rf = C_dl_/C_s_, where the double-layer capacitance (C_dl_) corresponds to the slope of the double-layer charging current plotted against the scan rate. The specific capacitance of the metal oxide (C_s_) was approximated as the average double-layer capacitance of a smooth oxide surface, which is approximately 60 μF cm^−2^. Scan rates were selected ranging from 10 mV s^−1^ to 100 mV s^−1^, with an interval of 20 mV s^−1^. In this study, C_dl_ was estimated by plotting ΔJ = (J_a_ – J_c_) at 0.8 V vs. RHE against the scan rate, with J_a_ and J_c_ representing the anodic and cathodic current densities, respectively. The linear slope is equivalent to twice of the C_dl_.

In the two-electrode solid electrolyte cell (cell configuration II and III), designed for generating pure HCOOH and HCOONa solutions, an Sustainion X37-50 grade FA AEM and a Nafion N115 CEM were employed for anion and cation exchange, respectively. PEEK sheet was sandwiched between AEM and CEM, and were securely pressed against GDL cathode and IrO_2_-Ti mesh, respectively. Styrene-divinylbenzene sulfonated copolymer (DOWEX 50WX-8) with a particle size of ~50 μm was dispersed in a 0.5 M Na_2_SO_4_ solution and ion-exchanged with Na^+^ for 24 h, subsequently serving as the SSE for proton conduction within the PEEK channel. Humidified CO_2_ gas was supplied to cathode titanium flow chamber. Ultrapure water was employed to release the generated HCOOH and HCOONa solution in the solid-state electrolyte.

**Product analysis.** The gas product analysis was conducted using a gas chromatograph (GC-136, INESA, China) equipped with a thermal conductivity detector and a flame photometric detector. Thermal conductivity detector coupled with JN. TDX-01 columns was used to analyze H_2_ and CO concentration. The liquid product was quantitatively analyzed using a 400 MHz 1H-NMR spectrometer (Bruker, Germany), employing the internal standard method. Typically, 600 μL of electrolyte obtained after the electrocatalytic process was mixed with 100 μL of D_2_O and 10 μL of 0.14 M DMSO, with DMSO serving as an internal standard. and the H in the formate is a single peak at about 8.3 ppm. The H in DMSO is a single peak at about 2.5 ppm. The faradaic efficiency (FE) for each product can be calculated using the following formula:

$$\text{f}\text{ \% = }\frac{\text{z}\text{ }\text{×}\text{ }\text{n}\text{ }\text{×}\text{ }\text{F}}{\text{Q}}\text{ ×100\%}$$

where z is the number of electrons required to form a molecular product, n is the amount of produced (mol), F is the Faraday's constant (96485.33 C mol^−1^), and Q is the total charge passed during the reaction (C).

**In situ ATR-FTIR measurement.** In situ ATR-FTIR spectroscopy was employed to detect intermediate products during the CO_2_RR. using a Thermo iS50 FT-IR spectrometer equipped with a liquid-nitrogen-cooled MCT-A detector. The in situ FTIR spectra were collected via internal reflection. The GB-Bi catalyst served as the working electrode, while an Ag/AgCl electrode and a platinum sheet electrode were used as the reference and counter electrodes, respectively. All measurements were conducted in a CO_2_-saturated 0.5 M Na_2_SO_4_ solution (pH adjusted to 2 with H_2_SO_4_). The applied potential was incremented positively from OCP to −1.5 V vs. RHE in 100 mV steps. In time-dependent measurements, a constant potential of −1.5 V vs. RHE was maintained, and spectra were acquired at 1, 3, 5, 10, 15, 20, 30, 40, 50, and 60 min. Upward bands in the spectra indicate the formation of products, whereas downward bands signify the consumption of reactants.^[1]^

**DFT computational details****.** Energy and structural optimizations were performed using DFT calculations, implemented in the CP2K code with the QUICKSTEP module and employing mixed Gaussian and plane-wave basis sets^[2]^. The electron interactions were modeled using the Perdew-Burke-Ernzerhof (PBE) functional within the generalized gradient approximation (GGA) framework. The core electrons of transition metal atoms were described using norm-conserving Goedecker–Teter–Hutter pseudopotentials. The valence electron wavefunctions were expanded in double-zeta basis sets with polarization functions, complemented by auxiliary plane-wave basis sets. An energy cutoff of 450 Ry was employed in all calculations. Each reaction intermediate structure was optimized by using the Broyden-Fletcher-Goldfarb-Shanno (BFGS) algorithm, with a self-consistent field (SCF) convergence criterion of 1.0×10^−5^ a.u. The electronic energy was augmented with the DFT-D3 scheme, which includes an empirical damping term to account for long-range dispersion interactions.^[3]^

In this study, the Gibbs free energy change (ΔG) for each elementary step in the CO_2_RR was calculated using the computational hydrogen electrode (CHE) model.

| $\Delta G={\Delta E}_{elec}+{\Delta E}_{ZPE}-T\Delta S+\Delta G_{pH}$ |
| --- |

where E_elec_ and E_zpe_ represent the electronic term, directly derived from DFT calculation, and the zero-point energy (ZPE) contribution, respectively. S denotes the entropy and T represents the temperature (298.15 K). ΔG_pH_ is the Gibbs free energy correction for the change in H+ concentration in the aqueous solution, where ΔG_pH_ = –k_B_ × T × ln(10 × pH). In this study, the pH was set at 2 to align with the central theme of the research.

Due to the minimal changes in translational and rotational entropies during the reaction, only the vibrational entropy contribution (S_vib_) was taken into account in this study.

| $S_{vib}=k_{B}\sum_{i} \left( \frac{hv_{i}}{k_{B}T\left( e^{\frac{hv_{i}}{k_{B}T}}-1 \right)}-ln\left( 1-e^{\frac{-hv_{i}}{k_{B}T}} \right) \right)$ |
| --- |

where k_B_ is the Boltzmann constant,$h$ is Planck’s constant and $v_{i}$ represents vibrational frequencies. The localized harmonic oscillator approximation, with a displacement of 0.01 Å, was employed for the vibrational frequency calculations. During the frequency calculation, only the reaction intermediates were allowed to relax, while all other atoms of the surface remained fixed.

**Analysis of contributions to cell voltage****.** The cell voltage (E_cell_) is composed of reversible cell voltage (E^0^) and the three main overpotentials (η).^[4]^

$$E_{cell}= E^{0} + \eta_{kin} + \eta_{ohm}+ \eta_{mass}$$

where η_kin_ is the kinetic overpotential, η_ohm_ is the ohmic overpotential, and η_mass_ is the mass transport overpotential.

In the CO_2_RR-to-formate process, the E^0^ of anode and cathode are presented below.^[5]^

Cathode reaction: ${CO}_{2}+2e^{-}+2H^{+}\to HCOOH E_{cathode}^{0}= -0.25 V vs.RHE$

Anode reaction: $2H_{2}O-4e^{-}\to O_{2}+4H^{+} E_{anode}^{0} =1.23 V vs.RHE$

Since, E^0^ can be obtained as E^0^ = 1.48 V.

EIS was used to measure the high frequency resistance (HFR) to represent the overall cell resistance R_ohm_. therefore, the ohmic losses of the cell η_ohm_ is determined as:

$$\eta_{ohm}=I \times R_{ohm}$$

η_kin_ was determined using a Tafel model, with the Tafel slope (b) and exchange current density (I_0_) as the key kinetic parameters. This model was fitted to IR-free water oxide reaction (WOR) and CO_2_RR electrolysis cell voltages at low current densities. The total η_kin_ for the cell is:

$$\eta_{\mathrm{kin}}=b \times\log\frac{I}{I_{0}}$$

Mass transport was defined as the sum of gaseous and liquid transfer in the gas diffusion layer (GDL) and catalyst layer (CL), as well as ionic transport in the CLs. The η_mass_ was calculated by subtracting the E^0^, η_kin_, and R_ohm_ from the E_cell_.

**TEA analysis of the CO_2_RR process**

The chemical equations for the formation of HCOONa and HCOOH are as follows:

For HCOONa production:

Cathode reaction: $2{CO}_{2}+4e^{-}+2H^{+}+2{Na}^{+}\to2HCOONa$

Anode reaction: $2H_{2}O-4e^{-}\to O_{2}+4H^{+}$

Overall reaction: ${2CO}_{2}+{2H}_{2}O +2{Na}^{+}\to2HCOONa+2H^{+}+O_{2}$

For HCOOH production:

Cathode reaction: $2{CO}_{2}+4e^{-}+4H^{+}\to2HCOOH$

Anode reaction: $2H_{2}O-4e^{-}\to O_{2}+4H^{+}$

Overall reaction: ${2CO}_{2}+{2H}_{2}O \to2HCOOH+O_{2}$

Therefore, the production of 1 mole of HCOOH necessitates the consumption of 1 mole of CO_2_ and 1 mole of H_2_O. instead, the production of 1 mole of HCOONa necessitates the consumption of 1 mole of CO_2_, 1 mole of H_2_O and 1 mole of Na^+^.

We assumed a cost analysis for the production of 100,000 kg of HCOONa/HCOOH per day with a capacity factor of 0.9 (32,850 tons per year), considering a factory lifespan of 20 years^[6]^.

According to long-term operation and SPCE test above, we assumed cell operation condition as Table S2.

**For HCOONa** production, the partial current needed is:

$$I_{HCOONa}=100,000\frac{kg}{day}*\frac{day}{86,400 s}*1,000\frac{g}{kg}*\frac{mol}{68.01}*2 e^{-}*96,485\frac{C}{mol}=3,284,001 A$$

The total current needed is the partial current divided by the faradaic efficiency:

$$I_{total}=\frac{3,284,001 A}{0.8}=4,105,001 A$$

The electrolyzer area needed is the total current divided by the current density:

$$Total electrolyzer area=\frac{4,105,001 A}{0.2 \frac{A}{{cm}^{2}}}*\frac{m^{2}}{10,000 {cm}^{2}}=2,053 m^{2}$$

The power needed is given from P=VI:

$$Power=2.8 V*4,105,001 A*\frac{W}{1,000 kW}=11,494 kW$$

The CO_2_ flow rate needed is:

$$CO_{2} flow=3,284,001 A*\frac{1}{2\frac{e^{-}}{mol CO_{2}}*96,485\frac{C}{mol}}*\frac{86,400 s}{day}=1,470,372 \frac{mol}{day}=64,696 \frac{kg}{day}$$

According to 80% single pass conversion efficiency of CO_2_, the inlet flow rate and outlet flow rate of CO_2_ are:

$$CO_{2} inlet flow=1,470,372\frac{mol}{day}*\frac{day}{24 h}*\frac{1}{0.8}=76,582 \frac{mol}{h}=3,370 \frac{kg}{h}$$

$$CO_{2} outlet flow=76,582 \frac{mol}{h}*\left( 1-0.8 \right)=15,316 \frac{mol}{h}=674 \frac{kg}{h}$$

$${CO}_{2} Outlet flow rate=\frac{{CO}_{2} outlet flow*R*T}{P}=\frac{15,316 \frac{mol}{hr}*8.314 \frac{J}{mol K}*298 K}{101 kPa}=375.7 \left( \frac{m^{3}}{h} \right)$$

It is assumed the byproduct is hydrogen (FE=20%), the flow rate of hydrogen is:

$$H_{2} production rate=4,105,001 A*3,600\frac{s}{h}*0.2*\frac{1}{2 e^{-}*96,485\frac{C}{mol}}=15,316 \frac{mol}{h}$$

$$H_{2} flow rate=\frac{H_{2} production rate*R*T}{P}=\frac{15,316 \frac{mol}{h}*8.314 \frac{J}{mol K}*298 K}{101 kPa}=375.7 \left( \frac{m^{3}}{h} \right)$$

The water flow rate for the anodic OER reaction is:

$$Anodic H_{2}O flow rate=4,105,001 A*3,600\frac{s}{h}*\frac{1}{4 e^{-}*96,485\frac{C}{mol}}*0.018\frac{kg}{mol}=689 \frac{kg}{h}$$

The liquid HCOONa product flow rate is:

$$liquid product flow rate=100,000\frac{kg}{day}*\frac{day}{24 h}*1,000\frac{g}{kg}*\frac{mol}{68.01}*\frac{1}{0.5 \frac{mol}{L}}=122,531 \frac{L}{h}$$

**For HCOOH** production, the partial current needed is:

$$I_{HCOOH}=100,000\frac{kg}{day}*\frac{day}{86,400 s}*1,000\frac{g}{kg}*\frac{mol}{46.03}*2 e^{-}*96,485\frac{C}{mol}=4,852,160 A$$

The total current needed is the partial current divided by the faradaic efficiency:

$$I_{total}=\frac{4,852,160 A}{0.7}=6,931,657 A$$

The electrolyzer area needed is the total current divided by the current density:

$$Total electrolyzer area=\frac{6,931,657 A}{0.2 \frac{A}{{cm}^{2}}}*\frac{m^{2}}{10,000 {cm}^{2}}=3,466 m^{2}$$

The power needed is given from P=VI:

$$Power=3.8 V*6,931,657 A*\frac{W}{1,000 kW}=26,340 kW$$

The CO_2_ flow rate needed is:

$$CO_{2} flow=4,852,160 A*\frac{1}{2\frac{e^{-}}{mol CO_{2}}*96,485\frac{C}{mol}}*\frac{86,400 s}{day}=2,172,496 \frac{mol}{day}=95,590 \frac{kg}{day}$$

According to 80% single pass conversion efficiency of CO_2_, the inlet flow rate and outlet flow rate of CO_2_ are:

$$CO_{2} inlet flow=2,172,496\frac{mol}{day}*\frac{day}{24 h}*\frac{1}{0.8}=113,151 \frac{mol}{h}=4,979 \frac{kg}{h}$$

$$CO_{2} outlet flow=113,151 \frac{mol}{h}*\left( 1-0.8 \right)=22,630 \frac{mol}{h}=996 \frac{kg}{h}$$

$${CO}_{2} Outlet flow rate=\frac{{CO}_{2} outlet flow*R*T}{P}=\frac{22,630 \frac{mol}{h}*8.314 \frac{J}{mol K}*298 K}{101 kPa}=555.1 \left( \frac{m^{3}}{h} \right)$$

It is assumed the byproduct is hydrogen (FE=30%), the flow rate of hydrogen is:

$$H_{2} production rate=6,931,657 A*3,600\frac{s}{h}*0.3*\frac{1}{2 e^{-}*96,485\frac{C}{mol}}=38,795 \frac{mol}{h}$$

$$H_{2} flow rate=\frac{H_{2} production rate*R*T}{P}=\frac{38,795 \frac{mol}{h}*8.314 \frac{J}{mol K}*298 K}{101 kPa}=951.7 \left( \frac{m^{3}}{h} \right)$$

The water flow rate for the anodic OER reaction is:

$$Anodic H_{2}O flow rate=6,931,657 A*3,600\frac{s}{h}*\frac{1}{4 e^{-}*96,485\frac{C}{mol}}*0.018\frac{kg}{mol}=1,164 \frac{kg}{h}$$

The liquid HCOONa product flow rate is:

$$liquid product flow rate=100,000\frac{kg}{day}*\frac{day}{24 h}*1,000\frac{g}{kg}*\frac{mol}{46.03}*\frac{1}{0.5 \frac{mol}{L}}=181,041 \frac{L}{h}$$

1. **Electolyzer cost**

We referenced the analysis re-calculated based on the stack cost from DOE Current Central H2A PEM electrolysis^[7]^. The reference electrolyzer cost for the stack component was 550 USD kW^−1^, and it operated at 1.75 V and 0.4 A cm^−2^. In comparison with our electolyzer, it was observed that the anodic water oxidation in PEM systems requires catalysts with a loading of 2 mg cm^−2^ of the precious iridium (Ir), which accounts for the dominant portion of the total electrode cost^[7a]^. Therefore, it can be inferred that the cost of our electolyzer should be similar to PEM model. To enable a more precise economic evaluation of diverse electrolytic cell configurations, we have recalculated the cost in terms of (USD m^−2^) rather than (USD kW^−1^), which facilitates a balanced comparison among electrolyzers with different power densities. According to the DOE H2A PEM model analysis, the electrolyzer cost per m^2^ can be calculated as below:

$$Electrolyzer {cost}_{\mathrm{per}m^{2}}=Reported cost \left( \frac{USD}{kW} \right)* Reported power density \left( \frac{kW}{m^{2}} \right)$$

$$=550\left( \frac{USD}{kW} \right)*\frac{0.4 A}{cm^{2}}*1.75V*\frac{kW}{10^{3} W}*\frac{10^{4} {cm}^{2}}{m^{2}}=3,850\frac{USD}{m^{2}}$$

According to the factory operation capacity factor of 0.9, which represents the ratio of practical production to the maximum potential production capacity, the electrolyzer cost can be calculated as below.

**For HCOONa:**

$$Electrolyzer cost=Total electrolyzer area*Electrolyzer {cost}_{\mathrm{per}m^{2}}*\frac{1}{capacity factor}$$

$$=2,053 m^{2}*3,850\frac{USD}{m^{2}}*\frac{1}{0.9}=8,782,278 USD$$

Assuming a capital discount rate (i) of 5%, the capital recovery factor (CRF) can be calculated as below:

$${CRF}_{electrolyzer}=\frac{{i(1+i)}^{lifetime}}{{(1+i)}^{lifetime}-1}=\frac{{0.05*(1+0.05)}^{20}}{{(1+0.05)}^{20}-1}=0.080$$

$${Electrolyzer cost}_{per ton HCOONa}=8,782,278 USD*\frac{0.080}{365}*\frac{1,000\frac{kg}{ton}}{100,000\frac{kg}{day}}=19.25 \frac{USD}{ton}$$

**For HCOOH:**

$$Electrolyzer cost=3,466 m^{2}*3,850\frac{USD}{m^{2}}*\frac{1}{0.9}=14,826,778 USD$$

$${Electrolyzer cost}_{per ton HCOOH}=14,826,778 USD*\frac{0.080}{365}*\frac{1,000\frac{kg}{ton}}{100,000\frac{kg}{day}}=32.50 \frac{USD}{ton}$$

1. **Balance of plant cost**

According to the DOE H2A analysis for distributed grid electrolysis, the Balance of Plant (BOP) cost, which including both mechanical components BoP and electrical BoP, is 39% of the total cost, while the stack is 61%. Thus, the total BoP capital cost is:

**For HCOONa:**

$$BoP cost=Electrolyzer cost \left( USD \right)*\frac{0.39}{0.61}=8,852,536 USD*\frac{0.39}{0.61}=5,659,818 USD$$

Herein, the BOP capital cost for production of per ton HCOONa can be calculated as below:

$${BoP cost}_{per ton HCOONa}=5,659,818 USD*\frac{0.080}{365}*\frac{1,000\frac{kg}{ton}}{100,000\frac{kg}{day}}=12.41 \frac{USD}{ton}$$

**For HCOOH:**

$$BoP cost=14,826,778 USD*\frac{0.39}{0.61}=9,479,415 USD$$

$${BoP cost}_{per ton HCOOH}=9,479,415 USD*\frac{0.080}{365}*\frac{1,000\frac{kg}{ton}}{100,000\frac{kg}{day}}=20.77 \frac{USD}{ton}$$

1. **Electricity cost**

We estimate the cost of electricity using the average industrial electricity price of US in 2024, which is 0.08 USD per kilowatt-hour as informed by data from the Department of Energy (DOE)^[8]^. To calculate the electricity cost per ton of formic acid, we divided the total electricity cost by the daily output of production.

**For HCOONa:**

$${Cost}_{electricity}=\frac{Power\times{24 \frac{h}{day}\times price}_{electricity}}{HCOONa production}=\frac{11,494 kW\times24 \frac{h}{day}\times\frac{0.08 USD}{kWh}}{100\left( \frac{ton HCOONa}{day} \right)}\boldsymbol{=}220.68 \frac{USD}{ton}$$

**For HCOOH:**

$${Cost}_{electricity}=\frac{26,340 kW\times24 \frac{h}{day}\times\frac{0.08 USD}{kWh}}{100\left( \frac{ton HCOOH}{day} \right)}\boldsymbol{=}505.73 \frac{USD}{ton}$$

1. **Pressure-swing adsorption (PSA) cost**

To enhance the recirculation of unreacted CO_2_, byproduct H_2_ is separated from the CO_2_ outlet stream. To estimate the costs of gaseous separation, we refer to data from a reference pressure-swing adsorption (PSA) system^[6-7]^.

**For HCOONa:**

$$PSA capital cost=reference cost*\left( \frac{total flow rate}{reference capacity} \right)^{scale factor}*\frac{1}{capacity factor}=1,989,043 USD*\left( \frac{375.7 \frac{m^{3}}{h} + 375.7 \frac{m^{3}}{h}}{1000 \frac{m^{3}}{h}} \right)^{0.7}*\frac{1}{0.9}=1,809,304 USD$$

$${PSA capital cost}_{per ton HCOONa}=1,809,304 USD*\frac{0.080}{365}*\frac{1,000 \frac{kg}{ton}}{100,000 \frac{kg}{day}}=3.97 \frac{USD}{ton}$$

$$PSA operating cost=\frac{total flow rate* reference PSA cost*electricity price}{target output production}=\frac{\left( 375.7 \frac{m^{3}}{h}+375.7 \frac{m^{3}}{h} \right)*24 \frac{h}{day}*0.25 \frac{kWh}{m^{3}}*0.08 \frac{USD}{kWh}}{100 \frac{ton HCOONa}{day}}=3.61 \frac{USD}{ton}$$

$$PSA total cost=PSA capital cost+PSA operating cost=7.58 \frac{USD}{ton}$$

**For HCOOH:**

$$PSA capital cost=1,989,043 USD*\left( \frac{555.1 \frac{m^{3}}{h} + 951.7 \frac{m^{3}}{h}}{1000 \frac{m^{3}}{h}} \right)^{0.7}*\frac{1}{0.9}=3,331,000 USD$$

$${PSA capital cost}_{per ton HCOOH}=3,331,000 USD*\frac{0.080}{365}*\frac{1,000 \frac{kg}{ton}}{100,000 \frac{kg}{day}}=7.30 \frac{USD}{ton}$$

$$PSA operating cost=\frac{\left( 555.1 \frac{m^{3}}{h} + 951.7 \frac{m^{3}}{h} \right)*24 \frac{h}{day}*0.25 \frac{kWh}{m^{3}}*0.08 \frac{USD}{kWh}}{100 \frac{ton HCOONa}{day}}=7.23 \frac{USD}{ton}$$

$$PSA total cost=14.53 \frac{USD}{ton}$$

1. **Liquid product separation cost**

Distillation is a prevalent method for separating liquid products within the scope of CO_2_RR, as evidenced by publications. In this study, the produced HCOONa solution can also be purified and separated through distillation and filtration to yield solid HCOONa, and the resulting condensate can be reused. Since we produce electrolyte-free HCOONa and HCOOH products, we suppose the distillation operating cost of our product is 50% compared to the mixed product^[9]^. However, conventional distillation techniques are inadequate for the practical separation of formic acid and water, attributable to the minimal boiling point difference of 0.8°C. Fang et al. proposed the method of pressurized extraction distillation for the purification and separation of the formic acid product, and two towers were used to produce 95% formic acid, which meets the market selling requirements^[10]^. The referenced energy consumptions of the two towers are 1097.22 and 50.11 kWh, respectively.

**For HCOONa:**

$$Distillation capital cost=Distillation ref cost*\left( \frac{Product flow rate \left( \frac{L}{h} \right)}{Distillation ref scale\left( \frac{L}{min} \right)*60\frac{min}{h}} \right)^{scale factor}=4,162,240 USD*\left( \frac{122,531 \frac{L}{h}}{1,000\left( \frac{L}{min} \right)*60\frac{min}{h}} \right)^{0.7}=6,861,095 USD$$

$${Distillation capital cost}_{per ton HCOONa}=6,861,095 USD*\frac{0.080}{365}*\frac{1,000 \frac{kg}{ton}}{100,000 \frac{kg}{day}}=15.04 \frac{USD}{ton}$$

$$Distillation operating cost= ref operating cost*\left( \frac{Product flow rate \left( \frac{L}{h} \right)}{Distillation ref scale\left( \frac{L}{min} \right)*60\frac{min}{h}} \right)^{scale factor}=32037.71 \frac{USD}{day}*\frac{1}{100\frac{ton}{day}}*\left( \frac{122,531 \frac{L}{h}}{1,000\left( \frac{L}{min} \right)*60\frac{min}{h}} \right)^{0.7}*0.5=264.06 \frac{USD}{ton}$$

$${Distillation Cost}_{per ton HCOONa}= 15.04 \frac{USD}{ton}+264.06 \frac{USD}{ton}=279.10 \frac{USD}{ton}$$

**For HCOOH:**

$$Distillation capital cost=Distillation ref cost*\left( \frac{Product flow rate \left( \frac{L}{h} \right)}{Distillation ref scale\left( \frac{L}{min} \right)*60\frac{min}{h}} \right)^{scale factor}=4,162,240 USD*\left( \frac{181,041 \frac{L}{h}}{1,000\left( \frac{L}{min} \right)*60\frac{min}{h}} \right)^{0.7}=9,017,063 USD$$

$${Distillation capital cost}_{per ton HCOOH}=9,017,063 USD*\frac{0.080}{365}*\frac{1,000 \frac{kg}{ton}}{100,000 \frac{kg}{day}}=19.76 \frac{USD}{ton}$$

$$Distillation operating cost= ref operating cost*\left( \frac{Product flow rate \left( \frac{L}{h} \right)}{Distillation ref scale\left( \frac{L}{min} \right)*60\frac{min}{h}} \right)^{scale factor}=32037.71 \frac{USD}{day}*\frac{1}{100\frac{ton}{day}}*\left( \frac{181,041 \frac{L}{h}}{1,000\left( \frac{L}{min} \right)*60\frac{min}{h}} \right)^{0.7}*0.5=347.03 \frac{USD}{ton}$$

$$Liquid separation operational cost=\left( 1097.22 kW+50.11 kW \right)*24 \frac{h}{day}*\frac{1}{100 \frac{ton}{day}}*0.08 \frac{USD}{kWh}=22.02 \frac{USD}{ton}$$

$${Liquid separation cost}_{per ton HCOOH}= 19.76 \frac{USD}{ton}+347.03 \frac{USD}{ton}+22.02 \frac{USD}{ton}=388.81 \frac{USD}{ton}$$

1. **Catalyst and membrane cost**

As mentioned above, the Ir catalyst used for the anodic water oxidation reaction (WOR) constitutes a significant portion of the electrolyzer cost. It is assumed that the costs of the catalyst and membrane account for 50% of the total electrolyzer cost^[10]^.

$${CRF}_{C\&M}=\frac{i{(1+i)}^{lifetime}}{{(1+i)}^{lifetime}-1}=\frac{0.07{(1.07)}^{5}}{{(1.07)}^{5}-1}=0.24389$$

**For HCOONa:**

$${Cost}_{C\&M}=\frac{{CRF}_{C\&M}*{Cost}_{Total Electrolyzer} \left( USD \right)*50\%}{Capacity factor*365\left( \frac{days}{year} \right)*production\left( \frac{ton}{day} \right)}=\frac{0.24389*8,782,278 USD*50\%}{0.9*365\left( \frac{days}{year} \right)*100\left( \frac{ton}{day} \right)}=32.60 \frac{USD}{ton}$$

**For HCOOH:**

$${Cost}_{C\&M}=\frac{{CRF}_{C\&M}*{Cost}_{Total Electrolyzer} \left( USD \right)*50\%}{Capacity factor*365\left( \frac{days}{year} \right)*production\left( \frac{ton}{day} \right)}=\frac{0.24389*14,826,778 USD*50\%}{0.9*365\left( \frac{days}{year} \right)*100\left( \frac{ton}{day} \right)}=55.04 \frac{USD}{ton}$$

Considering the variability of operating conditions in real-world applications, we also calculated the worst-case scenario, assuming that the catalysts (except for the Ir catalyst, due to its stability in WOR) and membrane have a lifetime of 0.5 year. In this case, the cost of additional catalysts and membrane, excluding the Ir catalyst, is considered to be 5% of the total electrolyzer cost.^[10-11]^

$${CRF}_{C\&M}=\frac{i{(1+i)}^{lifetime}}{{(1+i)}^{lifetime}-1}=\frac{0.07{(1.07)}^{0.5}}{{(1.07)}^{0.5}-1}=2.1044$$

**For HCOONa:**

$$Additional {cost}_{C\&M}=\frac{{CRF}_{C\&M}*{Cost}_{Total Electrolyzer} \left( USD \right)*5\%}{Capacity factor*365\left( \frac{days}{year} \right)*production\left( \frac{ton}{day} \right)}=\frac{2.1044*8,782,278 USD*5\%}{0.9*365\left( \frac{days}{year} \right)*100\left( \frac{ton}{day} \right)}=28.13 \frac{USD}{ton}$$

**For HCOOH:**

$${Additional cost}_{C\&M}=\frac{{CRF}_{C\&M}*{Cost}_{Total Electrolyzer} \left( USD \right)*50\%}{Capacity factor*365\left( \frac{days}{year} \right)*production\left( \frac{ton}{day} \right)}=\frac{2.1044*14,826,778 USD*50\%}{0.9*365\left( \frac{days}{year} \right)*100\left( \frac{ton}{day} \right)}=47.49 \frac{USD}{ton}$$

The additional costs of catalysts and membranes account for 4.61% and 4.49% of the total production costs for HCOONa and HCOOH, respectively.

**For HCOONa:**

$${Cost}_{C\&M}=\frac{{CRF}_{C\&M}*{Cost}_{Total Electrolyzer} \left( USD \right)*50\%}{Capacity factor*365\left( \frac{days}{year} \right)*production\left( \frac{ton}{day} \right)}=\frac{0.02523*8,782,278 USD*50\%}{0.9*365\left( \frac{days}{year} \right)*100\left( \frac{ton}{day} \right)}=32.60 \frac{USD}{ton}$$

**For HCOOH:**

$${Cost}_{C\&M}=\frac{{CRF}_{C\&M}*{Cost}_{Total Electrolyzer} \left( USD \right)*50\%}{Capacity factor*365\left( \frac{days}{year} \right)*production\left( \frac{ton}{day} \right)}=\frac{0.24389*14,826,778 USD*50\%}{0.9*365\left( \frac{days}{year} \right)*100\left( \frac{ton}{day} \right)}=55.04 \frac{USD}{ton}$$

1. **Input chemical cost**

**For HCOONa:**

The cost of input CO_2_ can be calculated by multiply CO_2_ consumption by a market price for CO_2_ (40 USD ton^−1^).

$$Input {CO}_{2} cost= 40\frac{USD}{ton {CO}_{2}}*64,696 \frac{kg {CO}_{2}}{day}*\frac{ton}{1,000 kg}*\frac{1}{100\frac{ton HCOONa}{day}}=25.87 \frac{USD}{ton}$$

Water consumption can be approximated by the anodic H_2_O flow rate, given that the water from the cathodic liquid product flow rate is recyclable in the distillation process. Therefore, the cost of input H_2_O can be calculated by multiply water consumption by a market price for water (5 USD ton^−1^).

$$Input H_{2}O cost=5\frac{USD}{ton H_{2}O}*689 \frac{kg H_{2}O}{h}*\frac{ton}{1,000 kg}*24\frac{h}{day}*\frac{1}{100\frac{ton HCOONa}{day}}=0.83 \frac{USD}{ton}$$

For HCOONa production, the electrolyte is 0.5 mol L^−1^ Na_2_SO_4_. As mentioned above, the production of 1 mole of HCOONa necessitates the consumption 1 mole of Na^+^, the electrolyte Na_2_SO_4_ cost can be calculated as:

$${Mass}_{{Na}_{2}{SO}_{4}} =1 ton HCOONa*\frac{1,000,000 g}{ton}*\frac{mol}{68.01 g}*\frac{142.04 g}{2 mol {Na}^{+}}*\frac{ton}{1,000,000 g}=1.044 \frac{ton}{ton HCOONa}$$

$${{Na}_{2}{SO}_{4} cost}_{per ton HCOONa}=1.044 ton*70\frac{USD}{ton}=73.08 \frac{USD}{ton}$$

**For HCOOH:**

$${Cost}_{intput {CO}_{2}}= 40\frac{USD}{ton {CO}_{2}}*95,590 \frac{kg {CO}_{2}}{day}*\frac{1}{1000}*\frac{1}{100\frac{ton HCOONa}{day}}=38.24 \frac{USD}{ton}$$

$$Input H_{2}O cost=5\frac{USD}{ton H_{2}O}*1,164 \frac{kg H_{2}O}{h}*\frac{ton}{1,000 kg}*24\frac{h}{day}*\frac{1}{100\frac{ton HCOONa}{day}}=1.40 \frac{USD}{ton}$$

For HCOOH production, the electrolyte is 0.5 mol L^−1^ H_2_SO_4_, which can be recycled. Assuming electrolyte lifetime is one year, it can be calculated that the CRF is 1.07. By using a fixed volume ratio of 100 L of electrolyte per square meter of electrolyzer for WOR, we can find the total volume of electrolyte needed.

$${Volume}_{\mathrm{electrolyte}} =electrolyzer area \left( m^{2} \right)*100 \left( \frac{L}{m^{2}} \right)=3,466 m^{2}*100\frac{L}{m^{2}}=346,600 L$$

$${Mass}_{H_{2}{SO}_{4}} = {molarity}_{H_{2}{SO}_{4}}\left( \frac{mol}{L} \right)*{Volume}_{electrolyte} \left( L \right)\times molecular weight \left( \frac{g}{mol} \right)=0.5\frac{mol}{L}*346,600 L*98.08\frac{g}{mol}= 16,997,264 g=17.00 ton$$

$${H_{2}{SO}_{4} cost}_{per ton HCOOH}=\frac{{CRF}_{electrolyzer}*{Mass}_{H_{2}{SO}_{4}}*H_{2}{SO}_{4} price}{Capacity factor*365\left( \frac{days}{year} \right)*production\left( \frac{ton}{day} \right)}= \frac{1.07*17.00 ton*185\frac{USD}{ton}}{0.9*365\left( \frac{days}{year} \right)*100\left( \frac{ton}{day} \right)}=0.10 \frac{USD}{ton}$$

1. **Other operational cost**

Other operational cost, including labor and maintenance et al., are presumed to constitute 2.5% of the electrolyzer's cost per ton of CO_2_.

**For HCOONa:**

$${Other cost}_{per ton HCOOH}=19.25 \frac{USD}{ton}*0.025=0.48 \frac{USD}{ton}$$

**For HCOOH:**

$${Other cost}_{per ton HCOOH}=32.50 \frac{USD}{ton}*0.025=0.81 \frac{USD}{ton}$$

**Total cost for one ton of Product:**

By summing all the above costs, the cost for producing one ton of formic acid can be obtained:

**For HCOONa:**

$${Cost}_{HCOONa}= {Cost}_{electrolyzer}+{Cost}_{BoP}+{Cost}_{electricity}+{{Cost}_{PSA}+{Cost}_{Liquid product separation}+Cost}_{catalyst and membrane}+{Cost}_{{CO}_{2}}+{Cost}_{water}{+ Cost}_{Electrolyte{Na}_{2}{SO}_{4}}+{Cost}_{other}=19.25+12.41+220.68+7.58+217.39+32.60+25.87+0.83+73.08+0.48\mathbf{=}610.17 \frac{USD}{ton}$$

**For HCOOH:**

$${Cost}_{HCOONa}= {Cost}_{electrolyzer}+{Cost}_{BoP}+{Cost}_{electricity}+{{Cost}_{PSA}+{Cost}_{Liquid product separation}+Cost}_{catalyst and membrane}+{Cost}_{{CO}_{2}}+{Cost}_{water}{+ Cost}_{Electrolyte H_{2}{SO}_{4}}+{Cost}_{other}=32.50+20.77+505.73+14.53+388.81+55.04+38.24+1.40+0.10+0.81\mathbf{=}1057.93 \frac{USD}{ton}$$

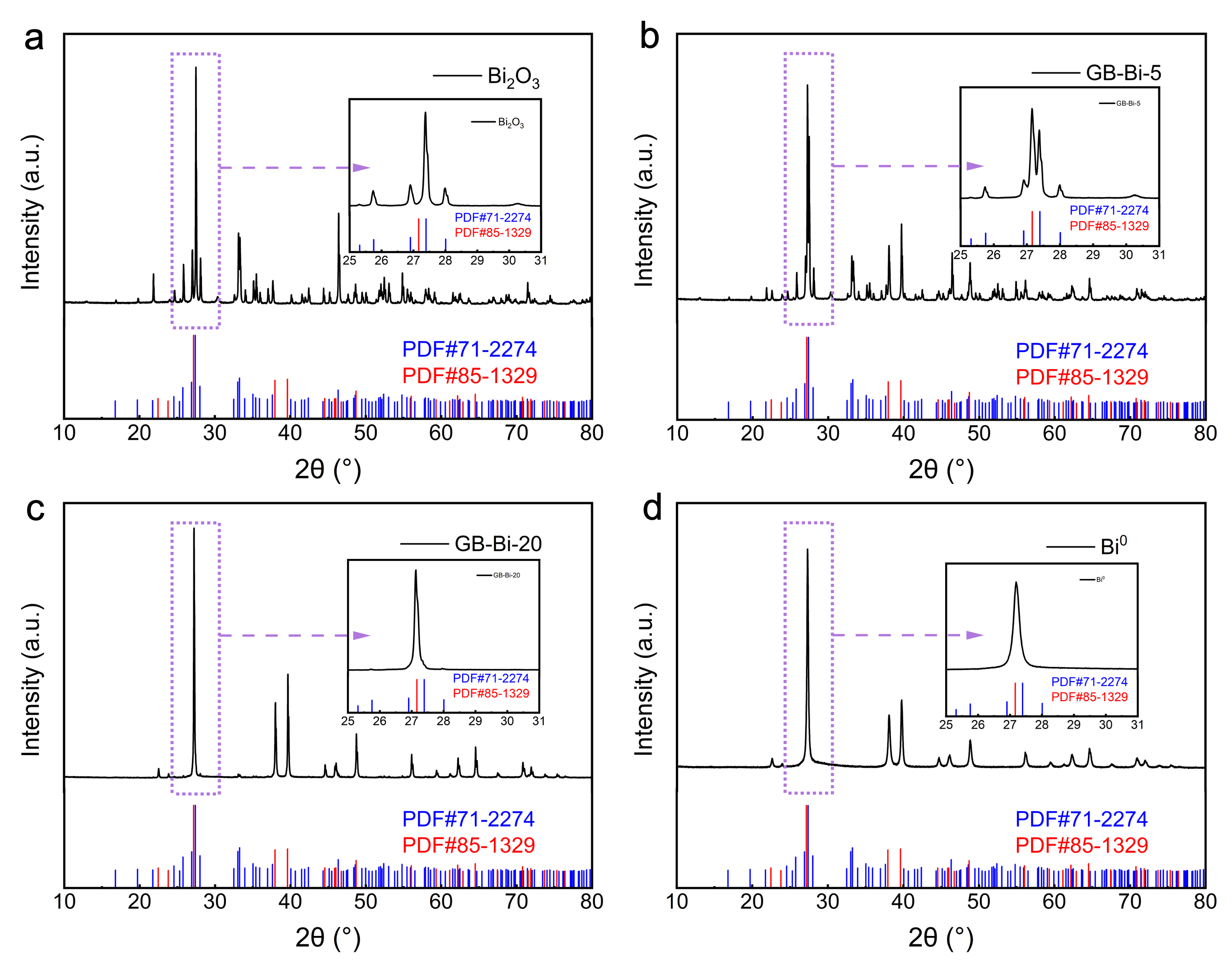


Figure S1. Detailed XRD pattern of a) Bi_2_O_3_, b) GB-Bi-5, c) GB-Bi-20, and d) Bi^0^.


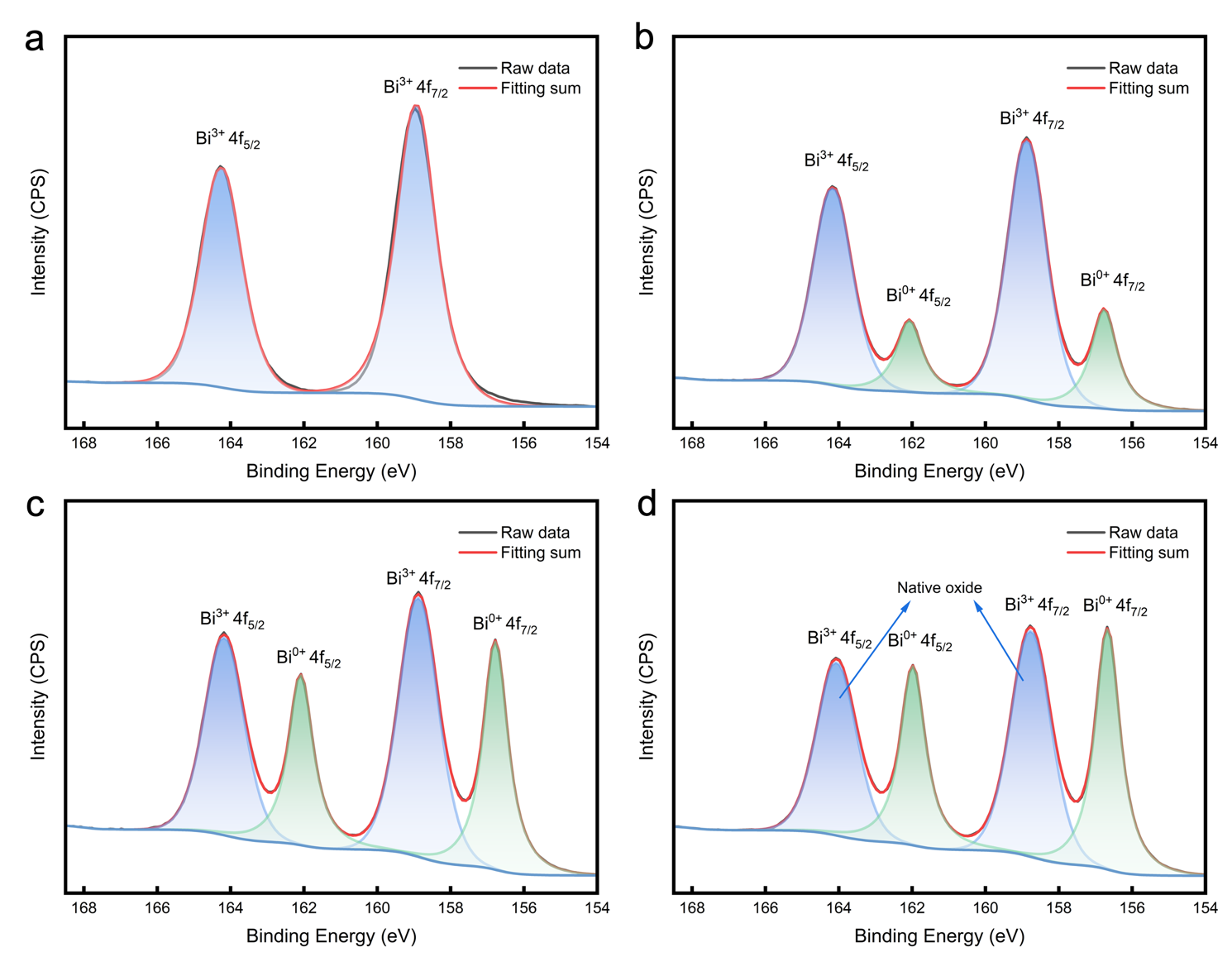


Figure S2. XPS spectra of a) Bi_2_O_3_, b) GB-Bi-5, c) GB-Bi-20, and d) Bi^0^.


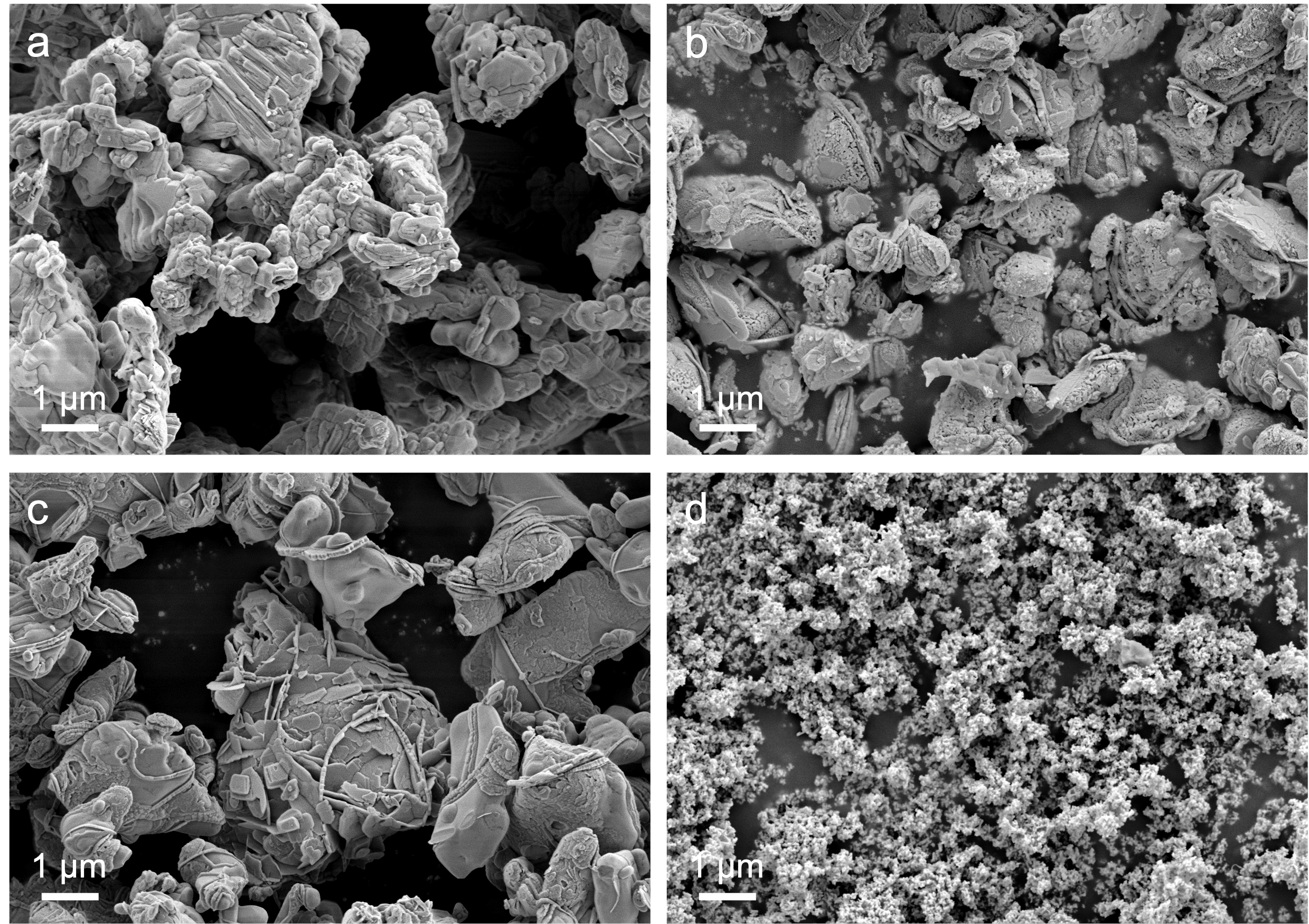


Figure S3. SEM images of a) Bi_2_O_3_, b) GB-Bi-5, c) GB-Bi-20, and d) Bi^0^ at ×10,000. Scale bar, 1 μm.


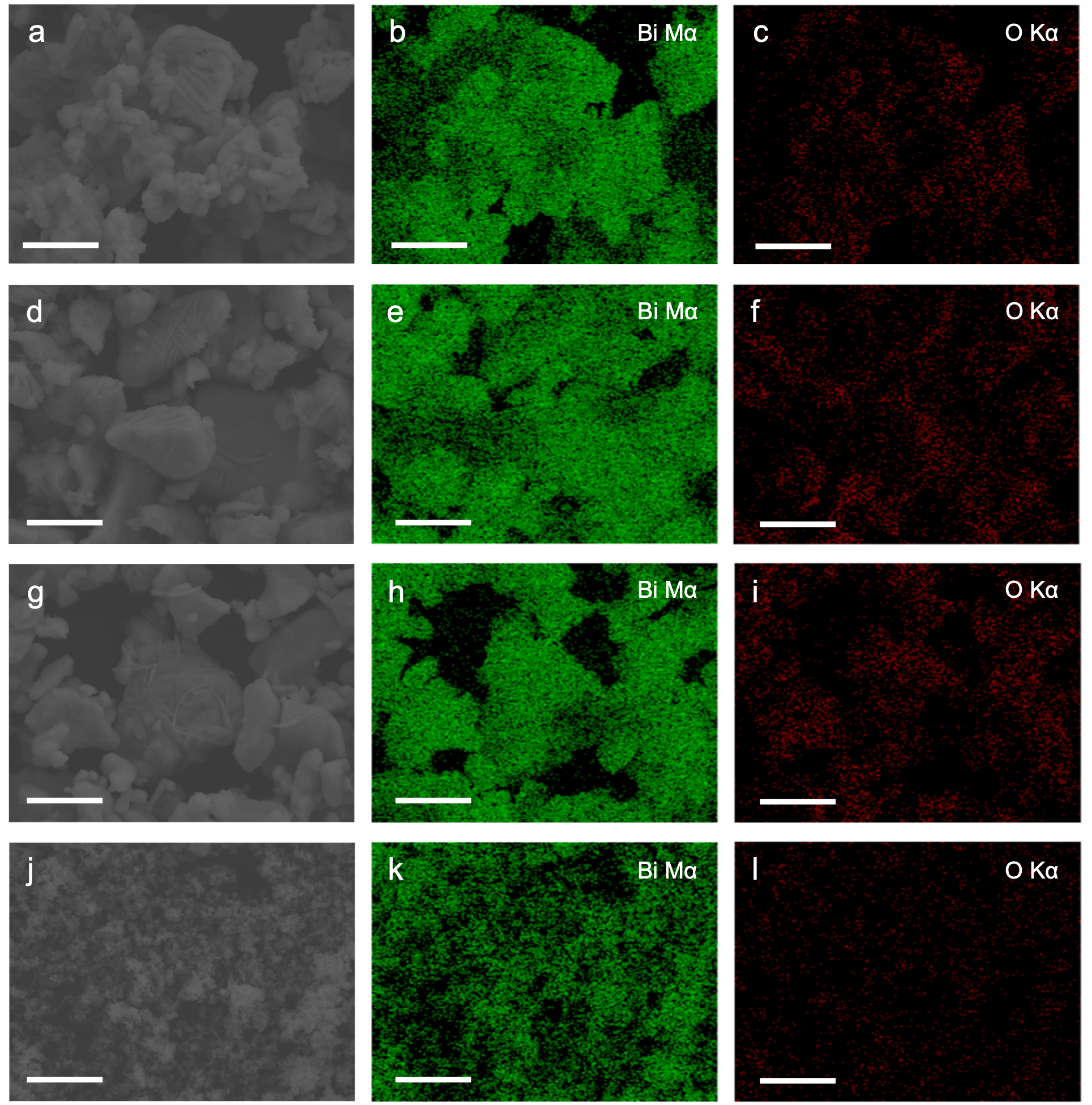


Figure S4. Mapping images of a-c) Bi_2_O_3_, d-f) GB-Bi-5, g-i) GB-Bi-20, and j-l) Bi^0^. Scale bar, 2.5 μm.


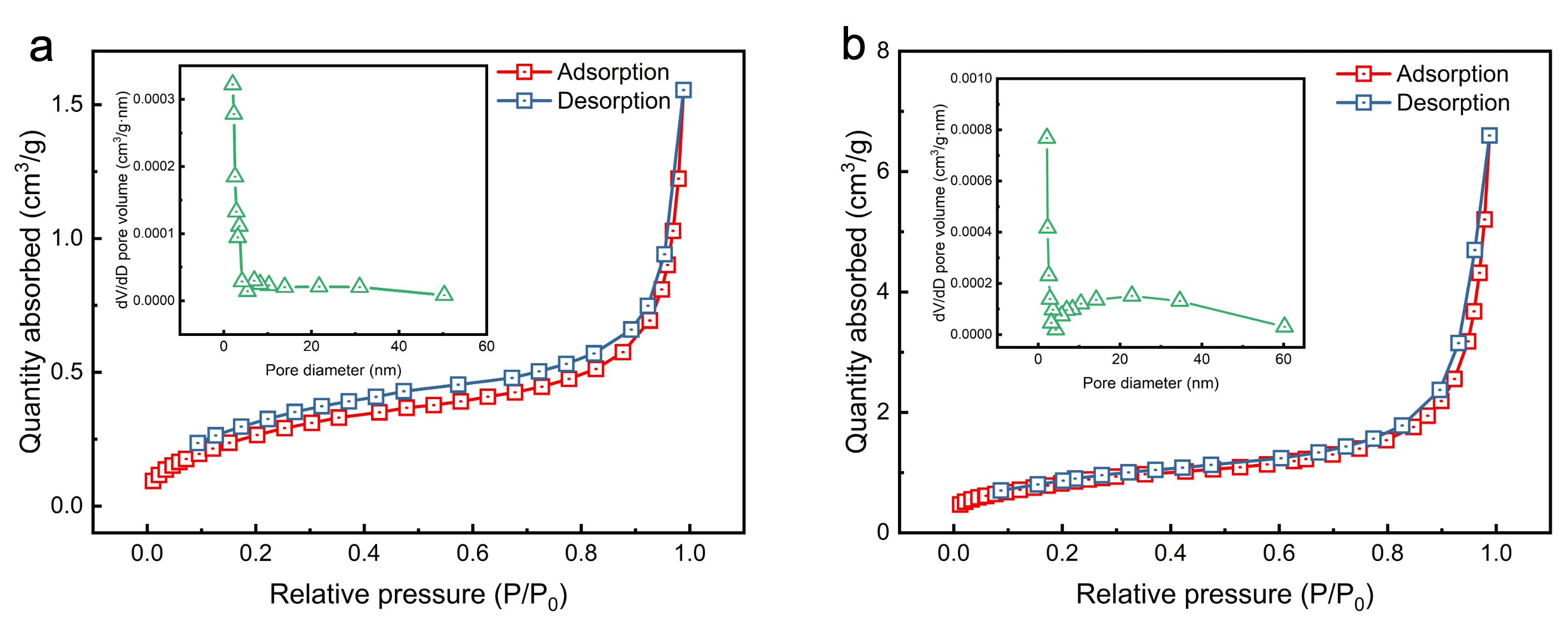


Figure S5. N_2_ adsorption/desorption isotherms and pore size distribution (the inset) of a) Bi_2_O_3_ and b) GB-Bi.


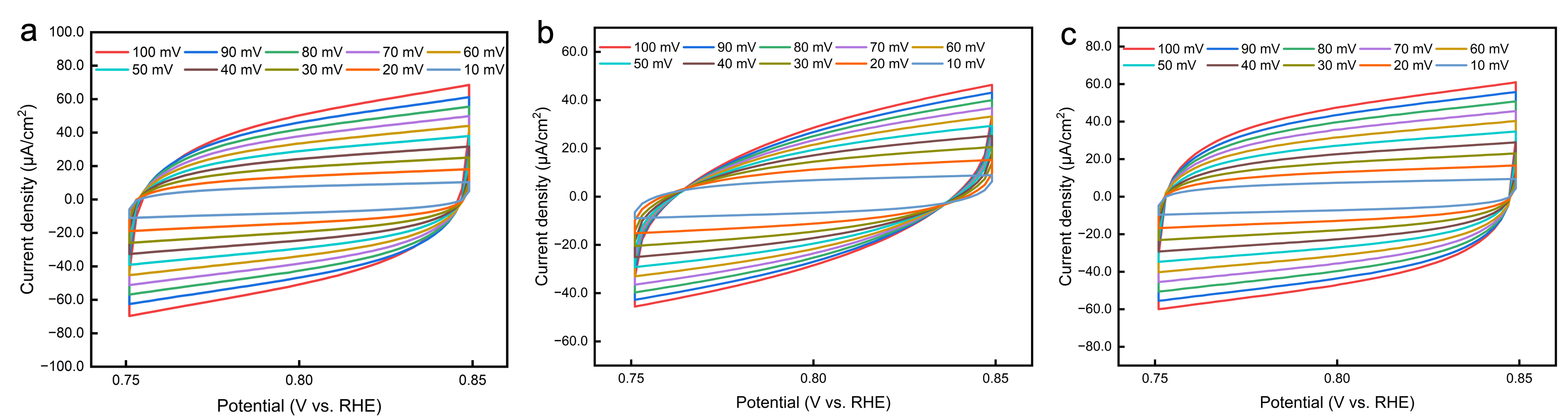


Figure S6. Cyclic voltammograms in the range of 0.75 to 0.85 V vs. RHE with different scan rates for (a) GB-Bi, (b) Bi_2_O_3_, and (c) Bi^0^.


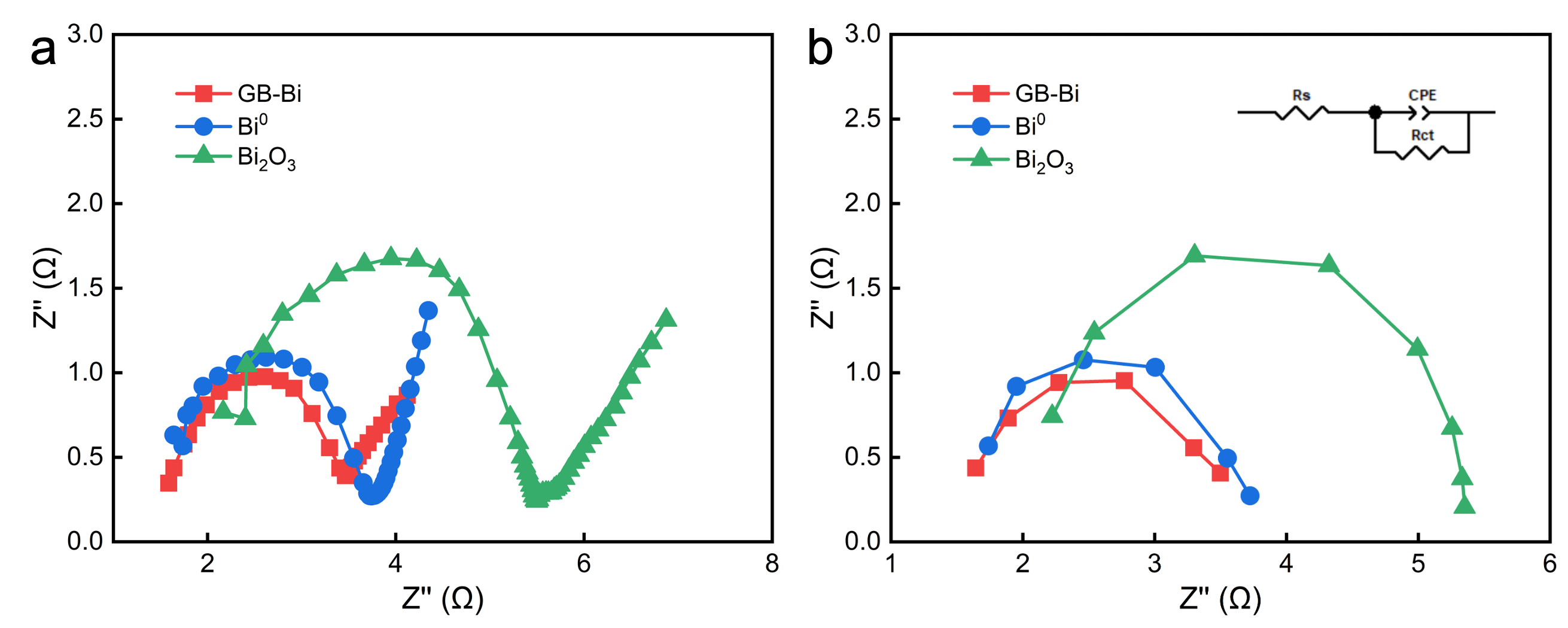


Figure S7. a) Nyquist plots and b) Nyquist plots fitted by the equivalent circuit (the inset) of GB-Bi, Bi_2_O_3_, and Bi^0^ at −0.9 V vs. RHE.


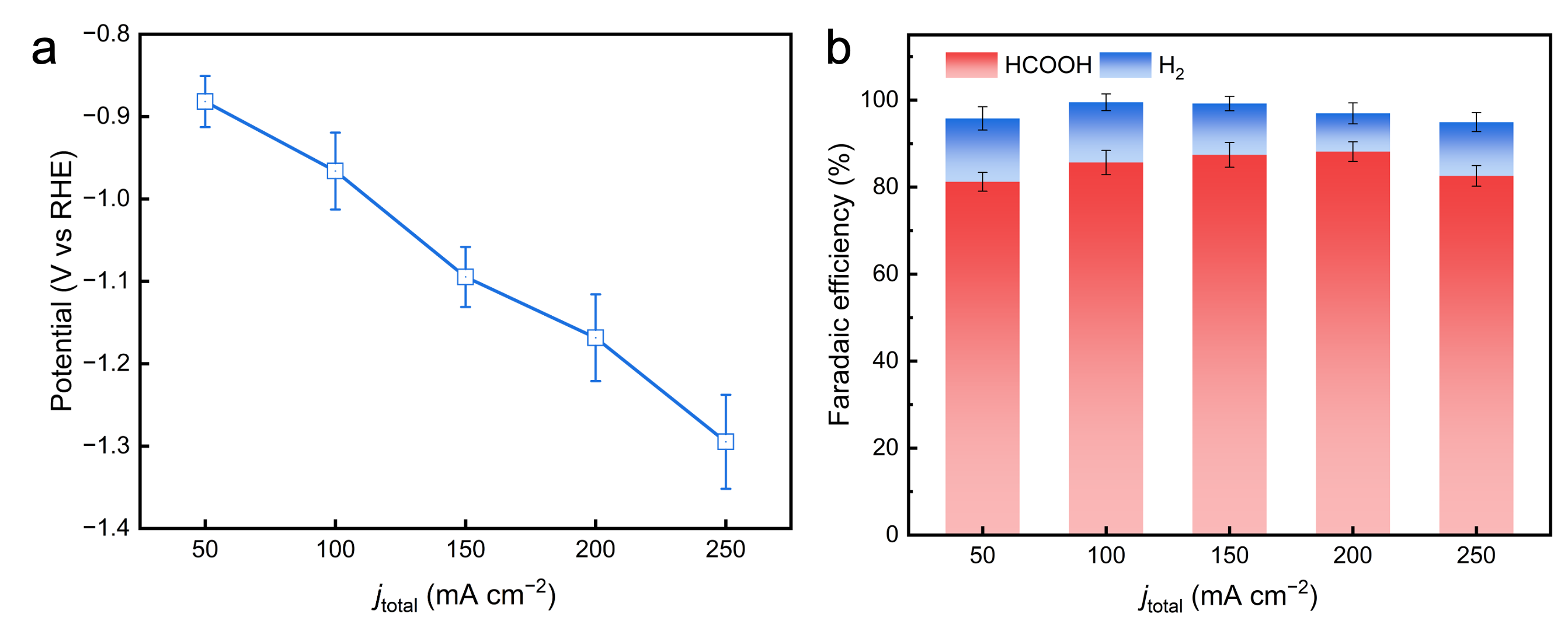


Figure S8. a) potential versus *j*_total_ curves of GB-Bi at acid condition and b) The corresponding FEs of different products at various current densities. A 0.5 M Na_2_SO_4_ solution at pH = 2 was used as electrolyte.


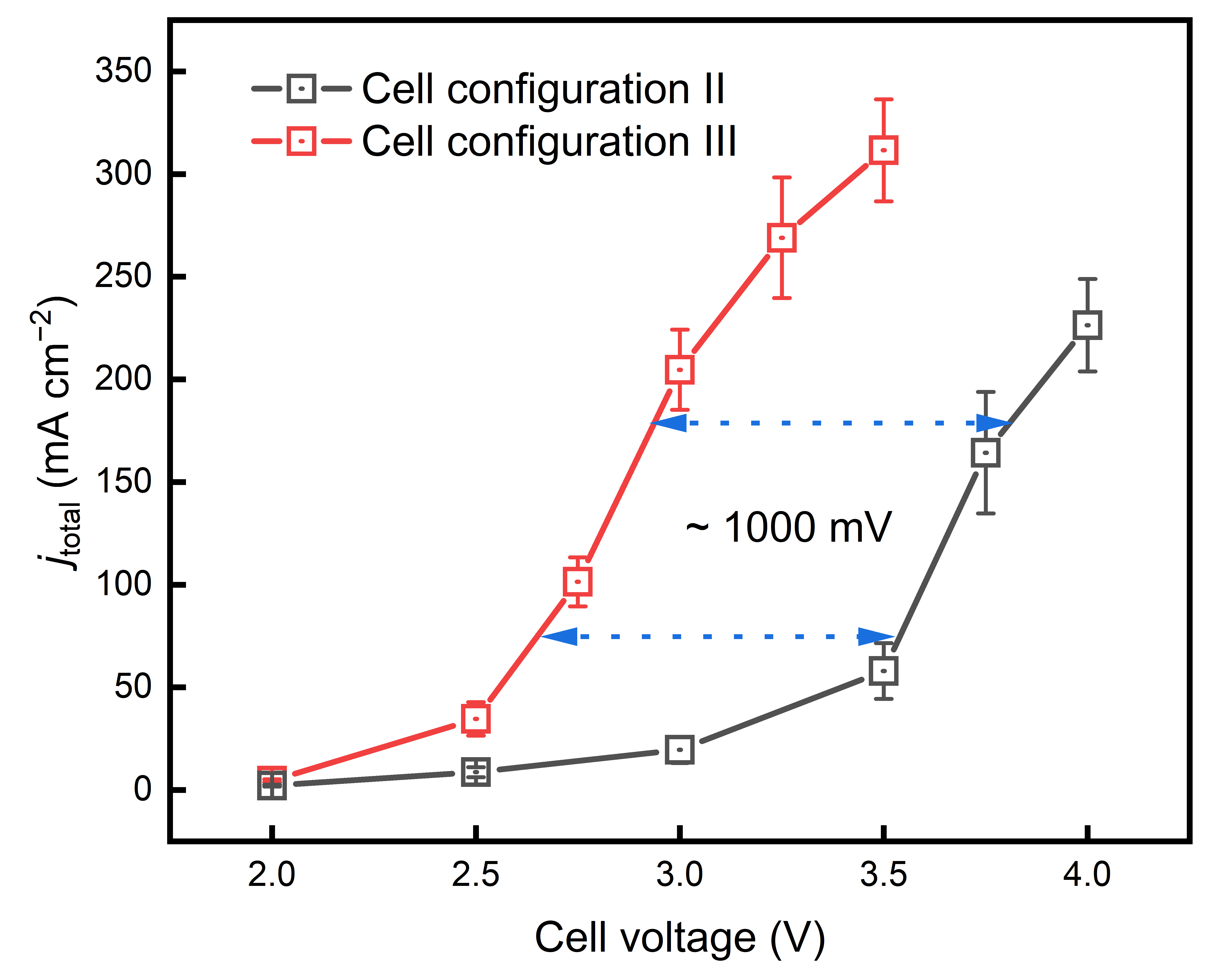


Figure S9. Comparison of *j*_total_–V curves in Cell Configuration II and Cell Configuration III, exhibiting a cell voltage reduction of ~1000 mV in HCOONa production process.


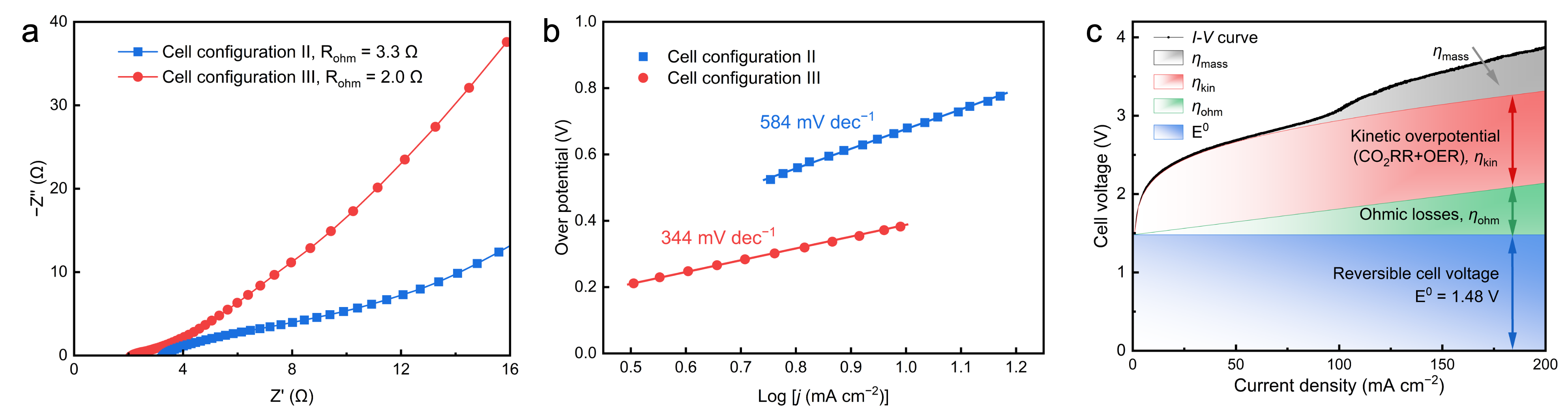


Figure S10. a) Nyquist plots in Cell Configuration II (R_ohm_ = 3.3 Ω) and Cell Configuration III (R_ohm_ = 2.0 Ω). b) Tafel plots of Cell Configuration II and Cell Configuration III. c) Individual contributions to cell voltage in Cell Configuration II, including reversible cell voltage, ohmic losses, kinetic overpotential and mass transport overpotential.


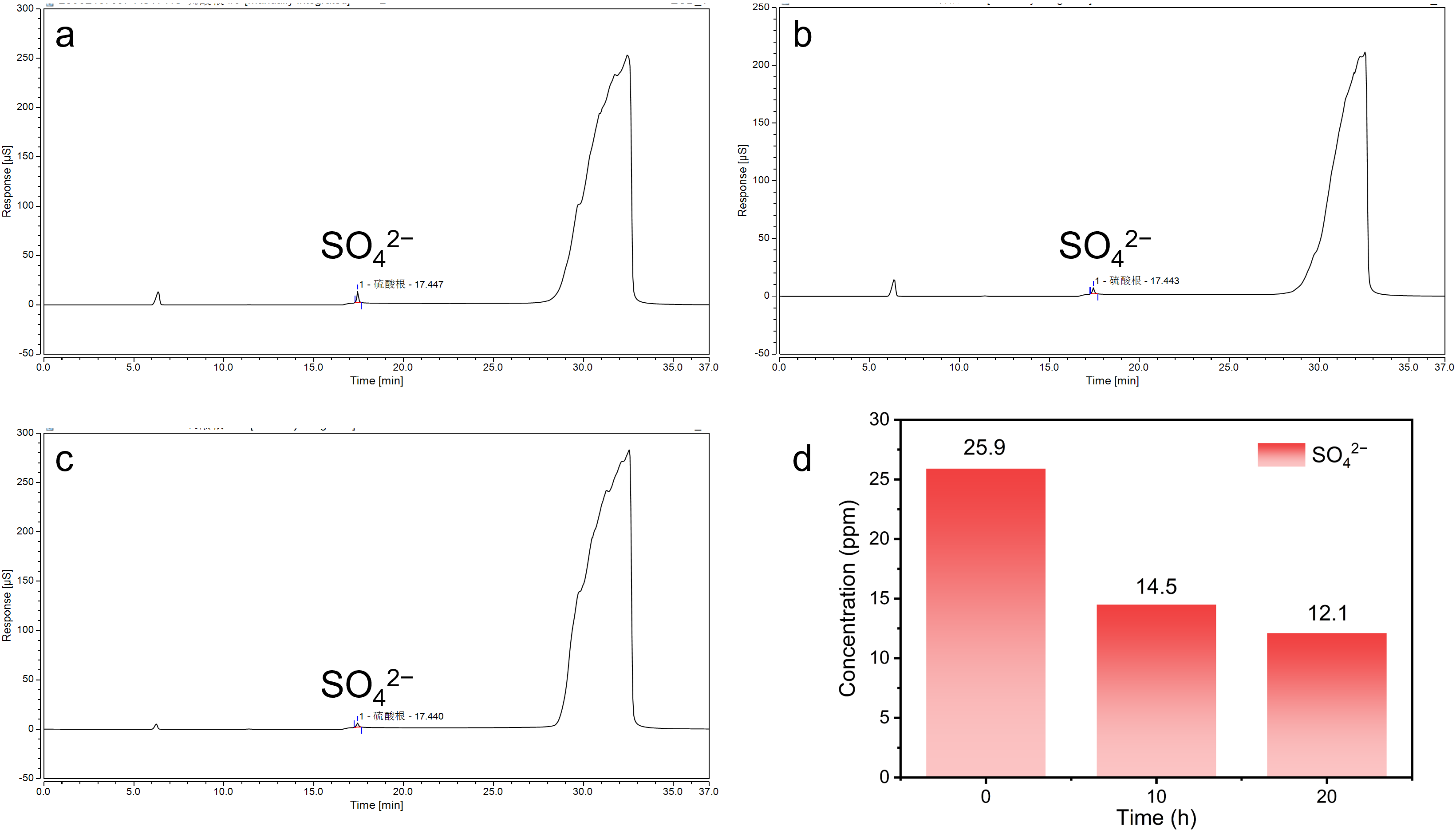


Figure S11. Ion chromatography results of sulfate ions in the electrolyte collected from SSE layer after different times of (a) 0 h, (b) 10 h and (c) 20 h using 0.5 M Na_2_SO_4_ (pH=1) as the anolyte. (d) The ion concentrations of sulfate vary over time.


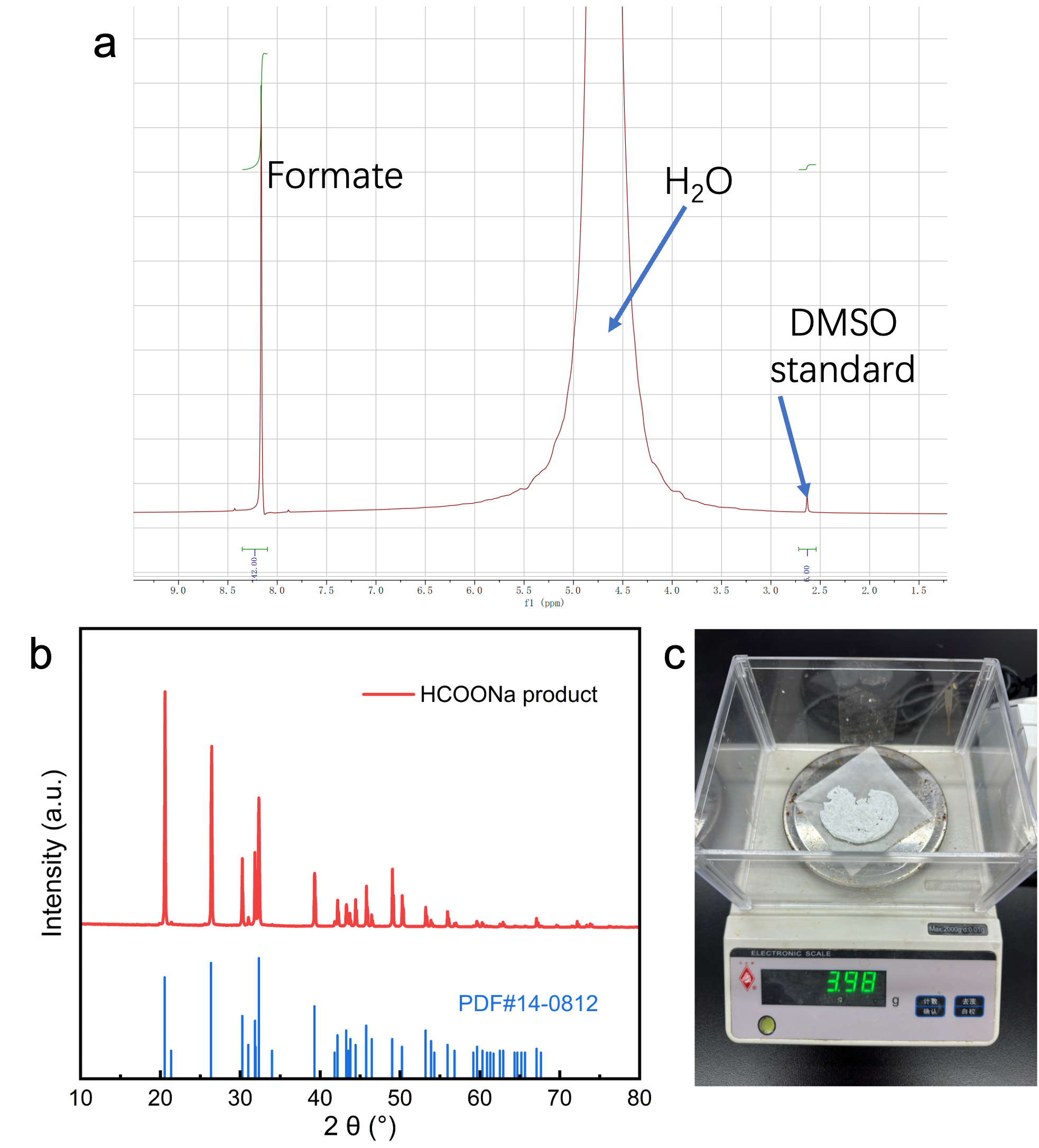


Figure S12. a) ^1^H-NMR spectrum of the pure HCOONa solution produced from MEA-SSE cell, b) XRD pattern of concentrated HCOONa precipitate obtained via an evaporation process. and c) concentrated 3.98 g HCOONa precipitate was obtained via an evaporation process after 24 h electrolysis.


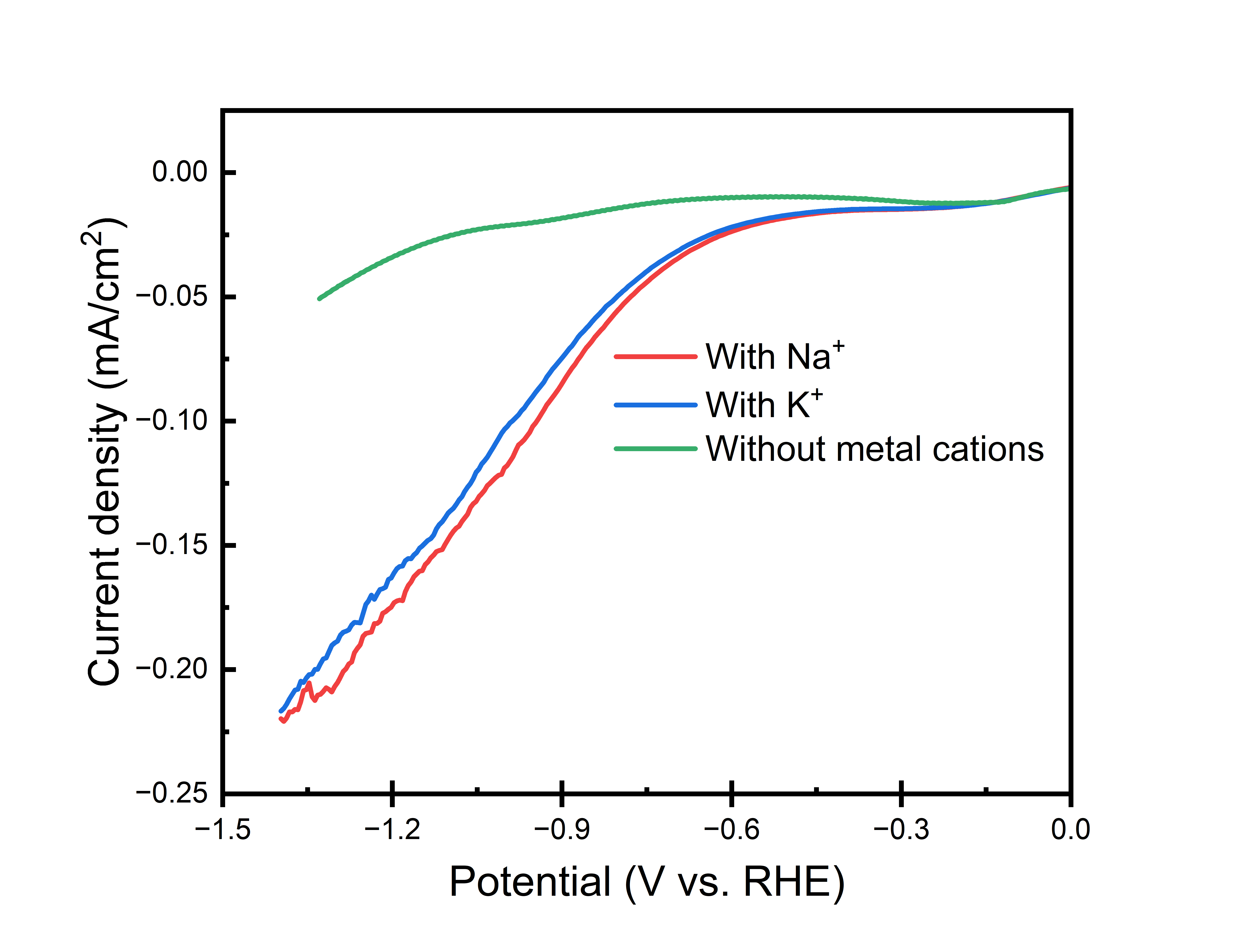


Figure S13. Liner sweep voltammograms of GB-Bi under different cations (1 M H^+^, 1M K^+^ at pH=1, 1M Na^+^ at pH=1), indicating that metal cations are crucial for the efficiency of CO_2_ electroreduction.


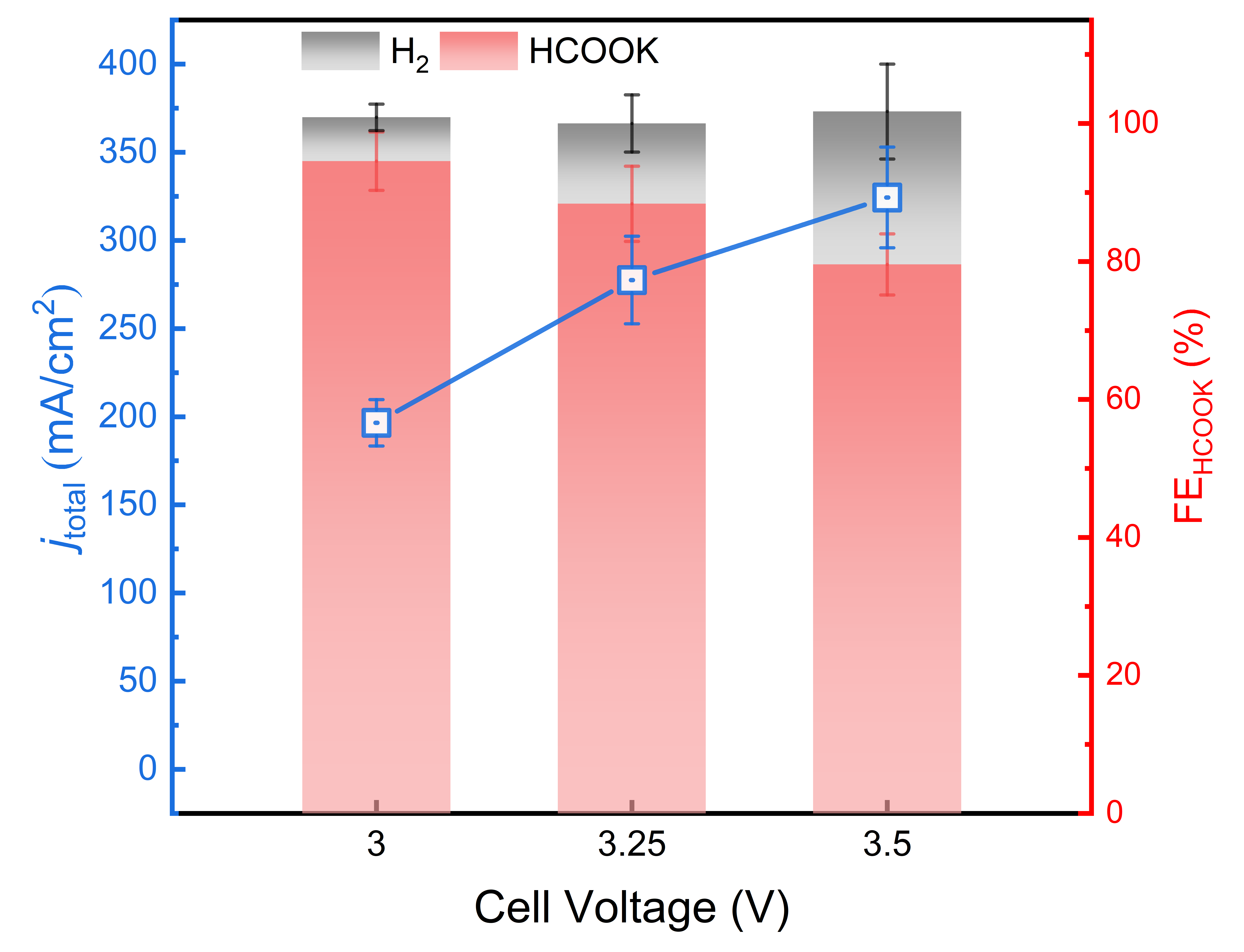


Figure S14. The *j*_total_–*V* curves and corresponding FEs of different cell voltages for HCOOK production in the MEA-SSE cell using 0.5 M K_2_SO_4_ (pH=1).


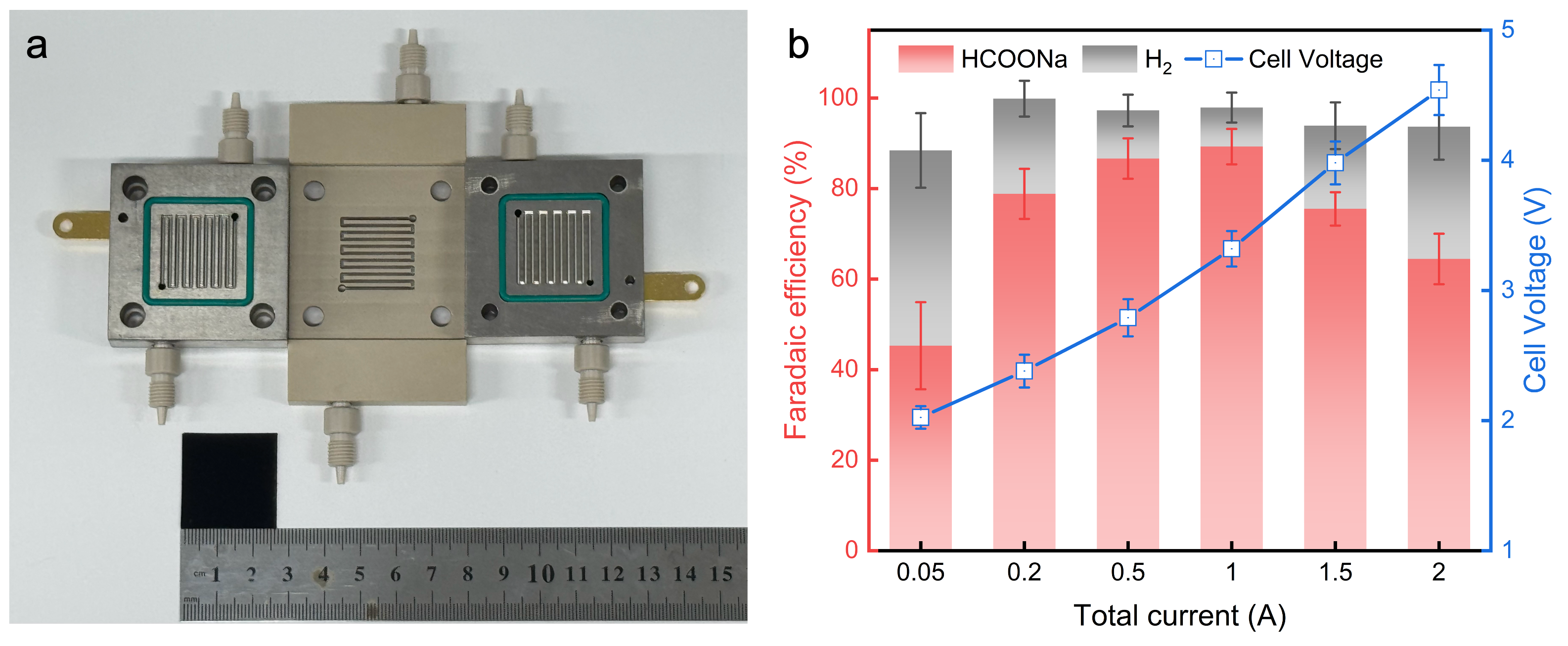
 Figure S15. a) Photograph of the MEA-SSE electrolyzer with an effective area of 4 cm^2^. b) The *I*_total_–*V* curves and corresponding FEs of different total current in a scaled-up MEA-SSE electrolyzer using 0.5 M Na_2_SO_4_ (pH=1.0).


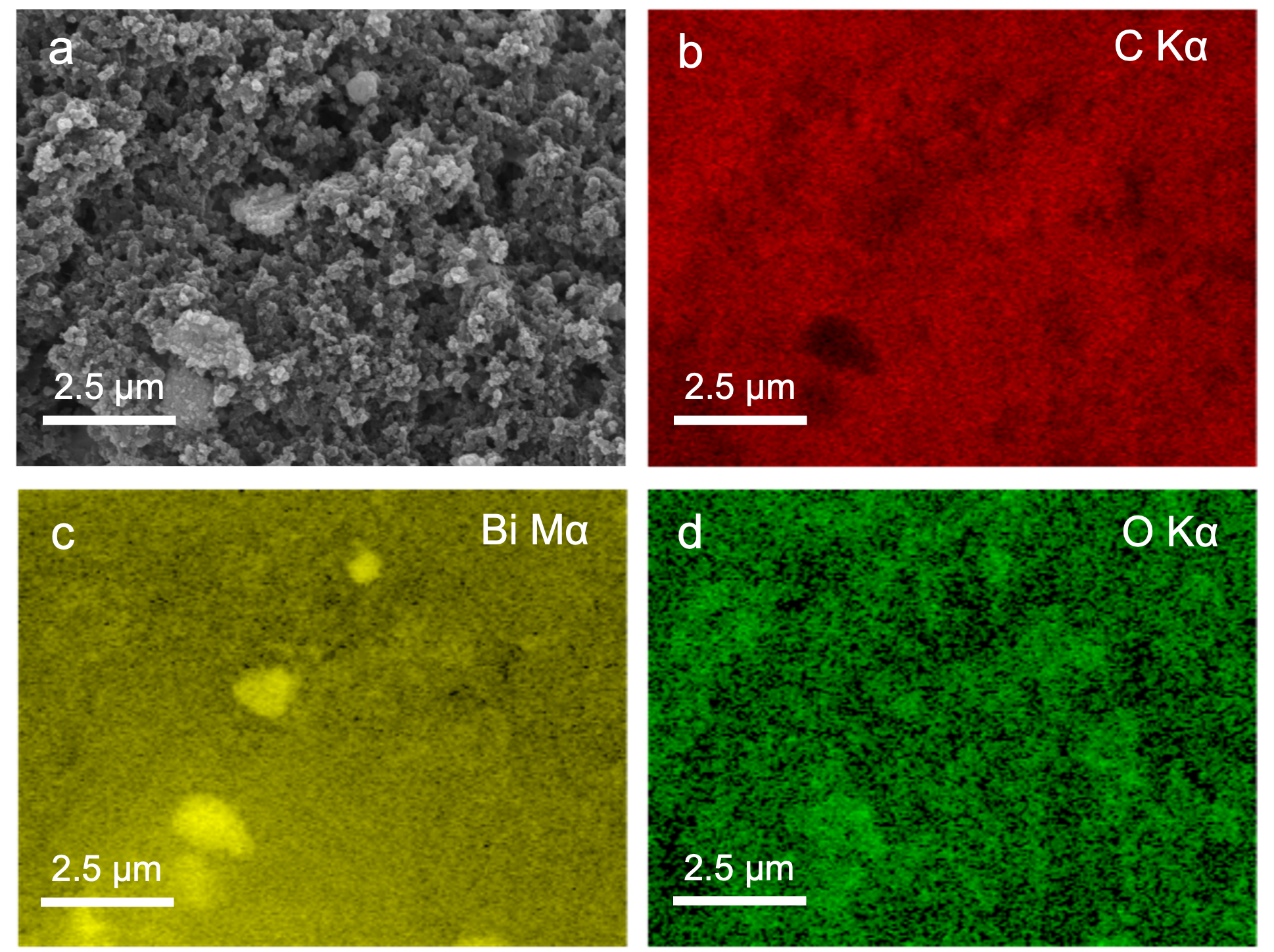


Figure S16. a) SEM images of surface view and it’s Mapping images of element b) C, c) Bi, and d) O of the cathode after CO_2_RR. Scale bar, 2.5 μm.


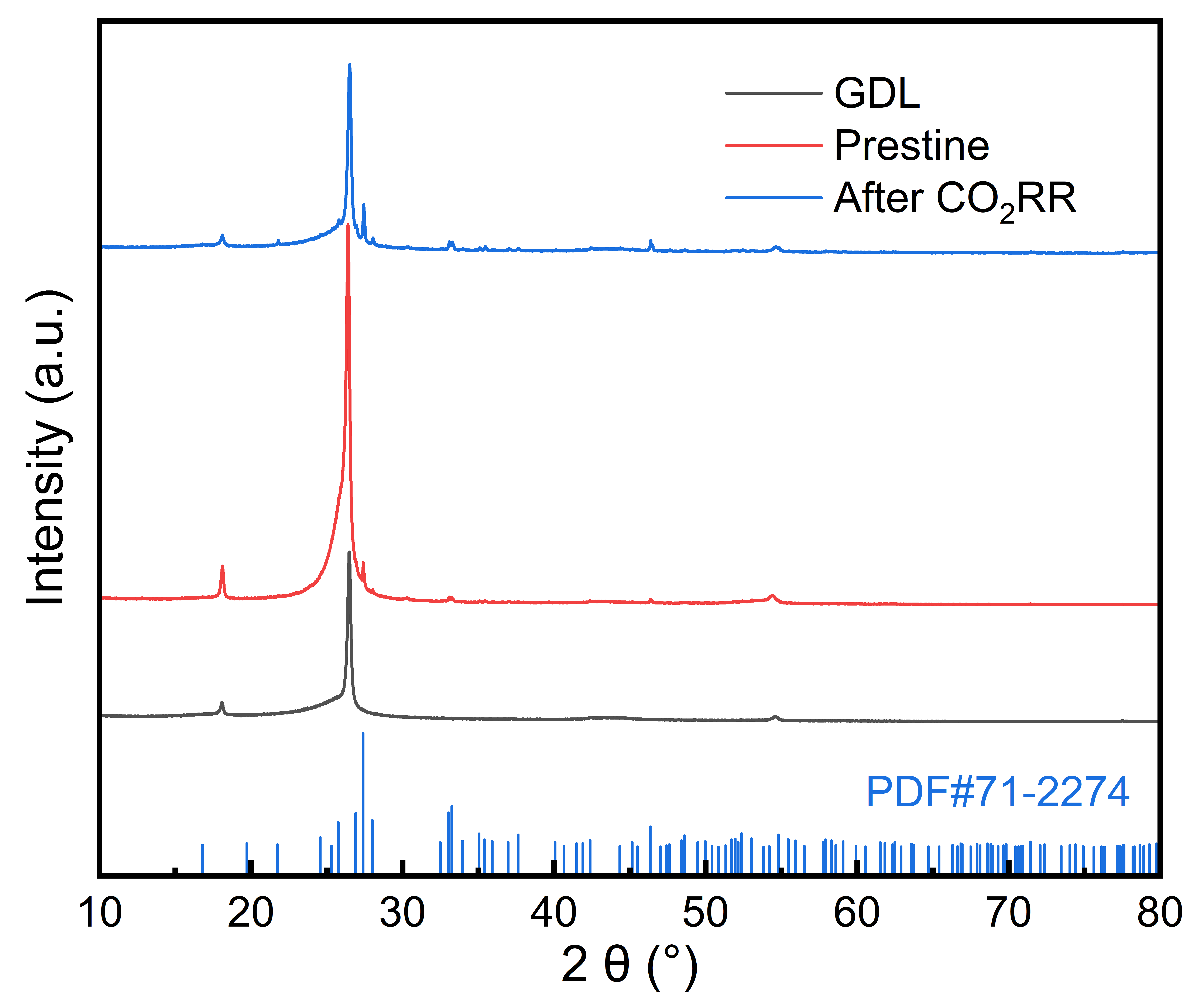


Figure S17. XRD pattern of GDL, pristine cathode and after electrolysis.


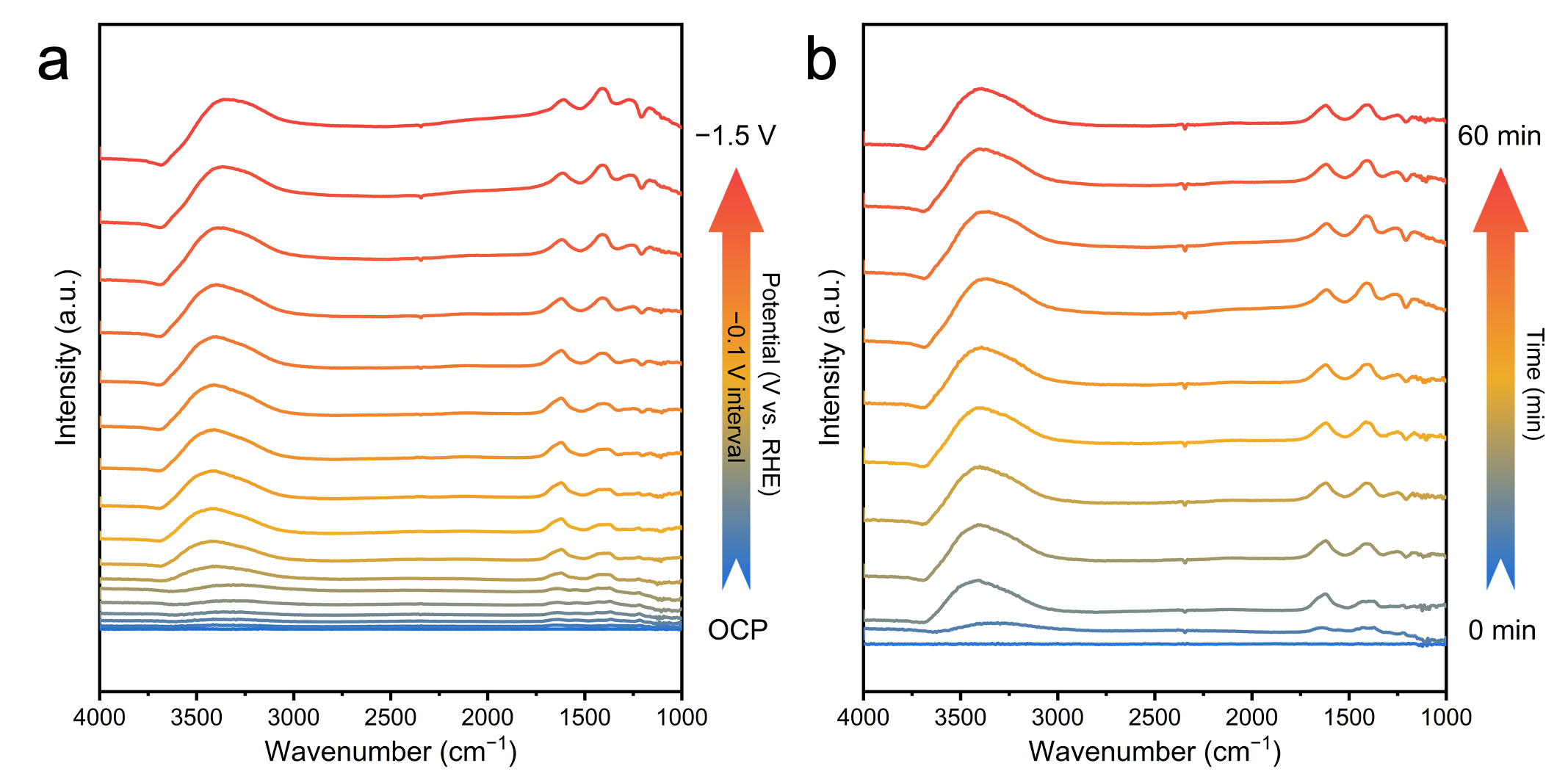


Figure S18. a) *In situ* ATR-FTIR spectra of full spectrum of GB-Bi under various potentials (vs. RHE) and b) Time-dependent *In situ* ATR-FTIR spectra of full spectrum of GB-Bi at −1.2 V vs. RHE. 0.5 M Na_2_SO_4_ solution at pH = 2.0 was used as electrolyte.


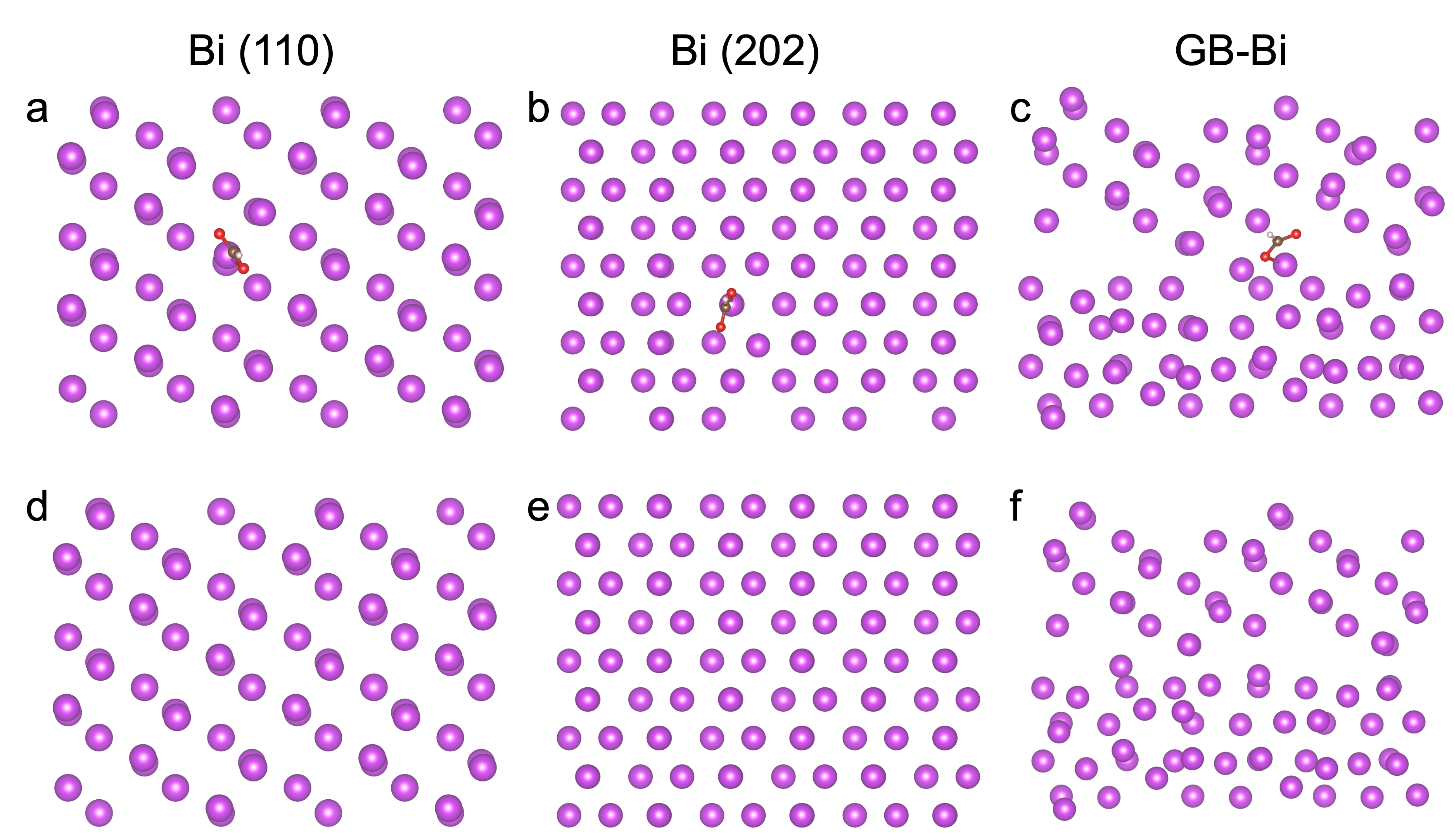


Figure S19. Top-views of the adsorption geometries of the intermediates *OCHO on a) Bi (110), b) Bi(202) and c) GB-Bi(110)/(202) surface. Top-views of d) Bi (110), e) Bi(202) and f) GB-Bi(110)/(202) model.


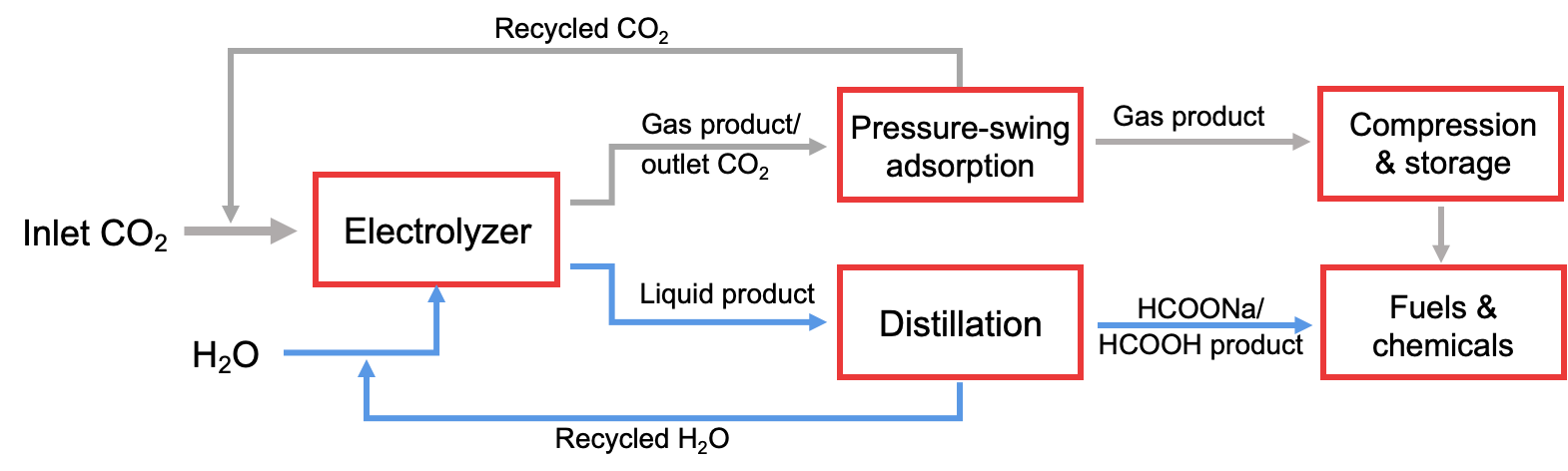


Figure S20. Techno-economic analysis framework of industrial grade CO_2_ electrolysis to HCOO^–^ process

**Table S1.** The concentration of different ions in HCOOH and HCOONa production using the MEA-SSE cell.

| Ions  Products | Bi | Na | K | Fe | Cu | Mg | Ca |
| --- | --- | --- | --- | --- | --- | --- | --- |
| HCOOH | <0.01 ppm | 3.26 ± 1.33 ppm | 0.60 ± 0.05 ppm | <0.01 ppm | <0.01 ppm | <0.01 ppm | <0.01 ppm |
| HCOONa | <0.01 ppm | 5.97 ± 0.73 g L^−1^ | <0.01 ppm | <0.01 ppm | <0.01 ppm | <0.01 ppm | <0.01 ppm |

Note: Concentrations of <0.01 ppm indicated that the ion concentration was below the detection limit.

**Table S2.** Summary of state-of-the-art researches on CO_2_ electrocatalytic production of electrolyte-free HCOOH/HCOONa products in SSE electrolyzer.

| **Products** | **Catalyst** | **Current density (mA·cm^−2^)** | **Cell voltage (V)** | **FE (%)** | **Anode requirement** | **Products concentration (mol L^−1^)** | **Stability (h)** | **Reference** |
| --- | --- | --- | --- | --- | --- | --- | --- | --- |
| HCOOH | 2D-Bi | 30 | 3 | ~80% | H_2_O (WOR) | 0.1 | 100 | A1^[9]^ |
| HCOOH | 2D-Bi | 37.5 | 2.9 | ~80.9% | H_2_O (WOR) | 1.13 | 20 | A2^[9]^ |
| HCOOH | 2D-Bi | 200 | -- | ~80% | H_2_O (WOR) | 2 (using N_2_ flow) | -- | A3^[9]^ |
| HCOOH | 2D-Bi | 200 | -- | ~40% | H_2_O (WOR) | 12.1 (using N_2_ flow) | -- | A4^[9]^ |
| HCOOH | nBuLi-Bi | 30 | 1.4 V in 50 h, 2.4 V after 60 h | ~80% | H_2_ (HOR) | ~0.1 | 100 | B1^[12]^ |
| HCOOH | nBuLi-Bi | 30 | 1.5 | ~75% | H_2_ (HOR) | ~0.35 (using N_2_ flow) | 30 | B2^[12]^ |
| HCOOH | nBuLi-Bi | 200 | 1.49 | 72% | H_2_ (HOR) | 2.4 (using N_2_ flow) | -- | B3^[12]^ |
| HCOOH | nBuLi-Bi | 200 | -- | -- | H_2_ (HOR) | 14.8 (using N_2_ flow) | -- | B4^[12]^ |
| HCOOH | Co_0.05_-BOON | 100 | 3.2 | 72% | H_2_O (WOR) | 0.1 | 100 | C1^[13]^ |
| HCOOH | Co_0.05_-BOON | 150 | 3.3 | 90.6% | H_2_O (WOR) | -- | -- | C2^[13]^ |
| HCOOH | Co_0.05_-BOON | 200 | 3.4 | 89% | H_2_O (WOR) | -- | -- | C3^[13]^ |
| HCOOH | Pb_1_Cu | 100 | 3.45 | 85% | H_2_O (WOR) | 0.1 | 180 | D^[14]^ |
| HCOOH | In_2_O_3_@C | 30 | 3.6 | ~80% | H_2_O (WOR) | 0.12 | 3 | E^[15]^ |
| HCOOH | Ag/Sn-SnO_2_ NSs | 100 | ~3.8 | -- | H_2_O (WOR) | 0.12 | 200 | F1^[16]^ |
| HCOOH | Ag/Sn-SnO_2_ NSs | 100 | ~3.8 V at 10 h, ~4.5 V after 12h | -- | H_2_O (WOR) | 0.27 (using N_2_ flow) | 25 | F2^[16]^ |
| HCOOH | BiSbO_x_/C | ~70 | 3.3 | -- | H_2_O (WOR) | -- | 13.33 | G1^[17]^ |
| HCOOH | BiSbO_x_/C | 200 | 4.3 | ~80% | H_2_O (WOR) | -- | -- | G2^[17]^ |
| HCOOH | BS/VC | 50 | ~3.8 | ~80% | H_2_O (WOR) | -- | 120 | H1^[18]^ |
| HCOOH | BS/VC | 100 | ~5 | ~80% | H_2_O (WOR) | -- | 80 | H2^[18]^ |
| HCOOH | Na_3_CA-NPB | 100 | ~4 | ~80% | H_2_O (WOR) | -- | 45 | I^[19]^ |
| HCOOH | InNCN | ~125 | 3.14 | 80% | H_2_O (WOR) | -- | 160 | J^[20]^ |
| HCOOH | GB-Bi | 200 | 3.8 | 70% | H_2_O (WOR) | 0.22 | 200 | This work |
| HCOOH | GB-Bi | 200 | 4 | 53% | H_2_O (WOR) | 2.2 | -- | This work |
| HCOONa | GB-Bi | 200 | 2.8 | 80% | H_2_O (WOR), Na^+^ | 0.27 | 300 | This work |
| HCOONa | GB-Bi | 200 | 3 | 50% | H_2_O (WOR), Na^+^ | 1.7 | -- | This work |

**Table S3.** Parameters of the Techno-economic analysis model

| **Input Costs** | **Value** | **Source** |
| --- | --- | --- |
| CO_2_ (USD ton^−1^) | 40.00 | ^[6]^ |
| Water (USD ton^−1^) | 5 | ^[21]^ |
| Electricity (USD kWh^−1^) | 0.08 | ^[8]^ |
| Onshore wind electricity (USD kWh^−1^) | 0.033 | ^[8, 22]^ |
| Na_2_SO_4_ (USD ton^−1^) | 70 | ^[23]^ |
| H_2_SO_4_ (USD ton^−1^) | 185 | ^[24]^ |
| Electrolyte Concentration (mol L^−1^) | 0.5 | This work |
| Target Production (ton day^−1^) | 100 | ^[6-7]^ |
| **Reactor Performance** | --- |  |
| Faradaic Efficiency (%) | 80% for HCOONa production  70% for HCOOH production | This work |
| Current Density (mA cm^−2^) | 200 | This work |
| Full Cell Voltage (V) | 2.8 V for HCOONa production  3.8 V for HCOOH production | This work |
| Single-Pass Carbon Utilization (%) | 80 | This work |
| Product Concentration (mol L^−1^) | 0.5 | This work |
| **Plant Parameters** | --- |  |
| Reference Electrolyzer Cost (USD kW^−1^) | 550 | ^[6, 25]^ |
| Reference Current Density (mA cm^−2^) | 400 | ^[6, 25]^ |
| Balance of Plant, BOP (%) | 50 | ^[11, 26]^ |
| Capacity Factor (%) | 90 | ^[10-11, 26a]^ |
| Electrolyzer Lifetime (years) | 20 | ^[10-11, 26a]^ |
| Catalyst + Membrane Lifetime (years) | 5 | ^[10-11, 26a]^ |
| Catalyst + Membrane/Electrolyzer Cost (%) | 50% | ^[10-11, 26a]^ |
| Electrolyte Lifetime (years) | 1 | ^[11, 26a]^ |
| Discount Rate (%) | 5 | ^[9, 26a]^ |
| Maintenance Frequency (years^−1^) | 5 | ^[10-11, 26a]^ |
| Maintenance Factor (%) | 15 | ^[10-11, 26a]^ |
| Anolyte Volume (L m^−2^) | 100 | ^[10, 26a]^ |
| **Gas Separation** | --- |  |
| PSA Operational Cost (kWh m^−3^) | 0.25 | ^[6-7, 10]^ |
| PSA Reference Capital Cost (USD) | 1 989 043.00 | ^[6-7, 10]^ |
| PSA Reference Capacity (m^3^ h^−1^) | 1000 | ^[6-7]^ |
| PSA Capacity Scaling Factor | 0.7 | ^[6-7]^ |
| **Liquid Separation** | --- |  |
| Distillation Reference Capital Cost (USD) | 4,162,240.00 | ^[6]^ |
| Distillation Reference Capacity (m^3^ h^−1^) | 1000 | ^[6]^ |
| Distillation Capacity Scaling Factor | 0.7 | ^[6, 10]^ |
| **Reference market price of product** | --- |  |
| HCOONa (USD ton^−1^) | 1050 | ^[27]^ |
| HCOOH (USD ton^−1^) | 900 | ^[28]^ |

**Table S4.** Range of values for sensitivity analysis

| **Sensitivity parameters** | **Pessimistic** | **Base** | **Optimistic** |
| --- | --- | --- | --- |
| Electricity price (USD kWh^−1^) | 0.11 | 0.08 | 0.033 |
| Product concentration (mol L^−1^) | 0.25 | 0.5 | 2.0 |
| Current density (mA cm^−2^) | 100 | 200 | 400 |
| Cell voltage (V) for HCOONa | 3.3 | 2.8 | 2.3 |
| Cell voltage (V) for HCOOH | 4.3 | 3.8 | 3.3 |
| Na_2_SO_4_ price (USD ton^−1^) | 120 | 70 | 40 |
| CO_2_ price | 70 | 40 | 0 |

**References**

[1] Y. Qin, H. Huang, W. Yu, H. Zhang, Z. Li, Z. Wang, J. Lai, L. Wang, S. Feng, Porous PdWM (M = Nb, Mo and Ta) trimetallene for high C1 selectivity in alkaline ethanol oxidation reaction. *Advanced Science* **2022**, *9*, 2103722.

[2] M. Krack, M. Parrinello, All-electron ab-initio molecular dynamics. *Physical Chemistry Chemical Physics* **2000**, *2*, 2105-2112.

[3] S. Grimme, J. Antony, S. Ehrlich, H. Krieg, A consistent and accurate ab initio parametrization of density functional dispersion correction (DFT-D) for the 94 elements H-Pu. *The Journal of Chemical Physics* **2010**, *132*, 154104.

[4] L. Wan, Z. Xu, Q. Xu, M. Pang, D. Lin, J. Liu, B. Wang, Key components and design strategy of the membrane electrode assembly for alkaline water electrolysis. *Energy & Environmental Science* **2023**, *16*, 1384-1430.

[5] Y. Zou, S. Wang, An investigation of active sites for electrochemical CO_2_ reduction reactions: from in situ characterization to rational design. *Advanced Science* **2021**, *8*, 2003579.

[6] M. Jouny, W. Luc, F. Jiao, General techno-economic analysis of CO_2_ electrolysis systems. *Ind. Eng. Chem. Res.* **2018**, *57*, 2165-2177.

[7] a) H. Shin, K. U. Hansen, F. Jiao, Techno-economic assessment of low-temperature carbon dioxide electrolysis. *Nat. Sustain.* **2021**, *4*, 911-919; b) D. Peterson, J. Vickers, D. DeSantis, DOE hydrogen and fuel cells program record. <https://www.hydrogen.energy.gov/pdfs/19009_h2_production_cost_pem_electrolysis_2019.pdf>

[8] Electricity data browser. <https://www.eia.gov/electricity/data/browser/#/topic/7>

[9] C. Xia, P. Zhu, Q. Jiang, Y. Pan, W. Liang, E. Stavitsk, H. N. Alshareef, H. Wang, Continuous production of pure liquid fuel solutions via electrocatalytic CO_2_ reduction using solid-electrolyte devices. *Nat. Energy* **2019**, *4*, 776-785.

[10] W. Fang, W. Guo, R. Lu, Y. Yan, X. Liu, D. Wu, F. M. Li, Y. Zhou, C. He, C. Xia, H. Niu, S. Wang, Y. Liu, Y. Mao, C. Zhang, B. You, Y. Pang, L. Duan, X. Yang, F. Song, T. Zhai, G. Wang, X. Guo, B. Tan, T. Yao, Z. Wang, B. Y. Xia, Durable CO_2_ conversion in the proton-exchange membrane system. *Nature* **2024**, *626*, 86-91.

[11] X. Wang, P. Ou, A. Ozden, S.-F. Hung, J. Tam, C. M. Gabardo, J. Y. Howe, J. Sisler, K. Bertens, F. P. García de Arquer, R. K. Miao, C. P. O’Brien, Z. Wang, J. Abed, A. S. Rasouli, M. Sun, A. H. Ip, D. Sinton, E. H. Sargent, Efficient electrosynthesis of n-propanol from carbon monoxide using a Ag–Ru–Cu catalyst. *Nat. Energy* **2022**, *7*, 170-176.

[12] L. Fan, C. Xia, P. Zhu, Y. Lu, H. Wang, Electrochemical CO_2_ reduction to high-concentration pure formic acid solutions in an all-solid-state reactor. *Nat. Commun.* **2020**, *11*, 3633.

[13] R. Nankya, Y. Xu, A. Elgazzar, P. Zhu, T.-U. Wi, C. Qiu, Y. Feng, F. Che, H. Wang, Cobalt-doped bismuth nanosheet catalyst for enhanced electrochemical CO_2_ reduction to electrolyte-free formic acid. *Angew. Chem. Int. Ed.* **2024**, *63*, e202403671.

[14] T. Zheng, C. Liu, C. Guo, M. Zhang, X. Li, Q. Jiang, W. Xue, H. Li, A. Li, C.-W. Pao, J. Xiao, C. Xia, J. Zeng, Copper-catalysed exclusive CO_2_ to pure formic acid conversion via single-atom alloying. *Nat. Nanotechnol.* **2021**, *16*, 1386-1393.

[15] Z. Wang, Y. Zhou, D. Liu, R. Qi, C. Xia, M. Li, B. You, B. Y. Xia, Carbon-confined indium oxides for efficient carbon dioxide reduction in a solid-state electrolyte flow cell. *Angew. Chem. Int. Ed.* **2022**, *61*, e202200552.

[16] M. Zhang, A. Cao, Y. Xiang, C. Ban, G. Han, J. Ding, L.-Y. Gan, X. Zhou, Strongly coupled Ag/Sn–SnO_2_ nanosheets toward CO_2_ electroreduction to pure HCOOH solutions at ampere-Level current. *Nano-Micro Lett.* **2023**, *16*, 50.

[17] X. Li, J.-H. Wang, C.-Y. Yuan, Q.-W. Sun, J. Shao, X.-C. Li, Z.-L. Feng, H. Dong, C. Li, Y.-W. Zhang, A unique amorphous porous BiSbO_x_ nanotube with abundant unsaturated Sb-stabilized BiO_8-x_ sites for efficient CO_2_ electroreduction in a wide potential window. *Adv. Funct. Mater.* **2024**, *34*, 2402220.

[18] J. Zhu, J. Li, R. Lu, R. Yu, S. Zhao, C. Li, L. Lv, L. Xia, X. Chen, W. Cai, J. Meng, W. Zhang, X. Pan, X. Hong, Y. Dai, Y. Mao, J. Li, L. Zhou, G. He, Q. Pang, Y. Zhao, C. Xia, Z. Wang, L. Dai, L. Mai, Surface passivation for highly active, selective, stable, and scalable CO_2_ electroreduction. *Nat. Commun.* **2023**, *14*, 4670.

[19] S. Guan, Z.-N. Chen, L.-Q. Liu, Y.-K. Li, C.-Y. Lan, J.-Z. Wang, P.-F. Yin, J. Yang, H. Liu, X.-W. Du, C. Dong, Nanoporous bismuth induced by surfactant-modified dealloying for efficient electrocatalytic reduction of CO_2_ to formic acid. *ACS Appl. Energ. Mater.* **2024**, *7*, 3201-3209.

[20] B. Jia, Z. Chen, C. Li, Z. Li, X. Zhou, T. Wang, W. Yang, L. Sun, B. Zhang, Indium cyanamide for industrial-grade CO_2_ electroreduction to formic acid. *J. Am. Chem. Soc.* **2023**, *145*, 14101-14111.

[21] B. S. Crandall, B. H. Ko, S. Overa, L. Cherniack, A. Lee, I. Minnie, F. Jiao, Kilowatt-scale tandem CO_2_ electrolysis for enhanced acetate and ethylene production. *Nat. Chem. Eng.* **2024**, *1*, 421-429.

[22] Levelized cost of energy by technology, United States. <https://ourworldindata.org/grapher/levelized-cost-of-energy?country=~USA>

[23] Industrial sodium sulfate ≥ 98%. https://4259eb70b35a9906.en.made-in-china.com/product/VQnUOTvjCwcY/ China-Industrial-Sodium-Sulfate-ge-98-.html?pv_id=1ibe23dlhb36&faw_id=1ibe23qqfd8b

[24] Industrial sulfuric acid 98%. <https://sjzxlwchem.en.made-in-china.com/product/ZvtxbWhAANcI/China-ISO-9001-2008-Certification-SGS-Test-Sulfuric-Acid.html?pv_id=1ibs2cbgcf17&faw_id=1ibs2cdocbf4>

[25] X. Jing, F. Li, Y. Wang, Assessing the economic potential of large-scale carbonate-formation-free CO_2_ electrolysis. *Catal. Sci. Technol.* **2022**, *12*, 2912-2919.

[26] a) J. Jin, J. Wicks, Q. Min, J. Li, Y. Hu, J. Ma, Y. Wang, Z. Jiang, Y. Xu, R. Lu, G. Si, P. Papangelakis, M. Shakouri, Q. Xiao, P. Ou, X. Wang, Z. Chen, W. Zhang, K. Yu, J. Song, X. Jiang, P. Qiu, Y. Lou, D. Wu, Y. Mao, A. Ozden, C. Wang, B. Y. Xia, X. Hu, V. P. Dravid, Y.-M. Yiu, T.-K. Sham, Z. Wang, D. Sinton, L. Mai, E. H. Sargent, Y. Pang, Constrained C2 adsorbate orientation enables CO-to-acetate electroreduction. *Nature* **2023**, *617*, 724-729; b) A. Ozden, Y. Wang, F. Li, M. Luo, J. Sisler, A. Thevenon, A. Rosas-Hernandez, T. Burdyny, Y. Lum, H. Yadegari, T. Agapie, J. C. Peters, E. H. Sargent, D. Sinton, Cascade CO_2_ electroreduction enables efficient carbonate-free production of ethylene. *Joule* **2021**, *5*, 706-719.

[27] Industrial grade sodium formate. <https://baoqu888.en.made-in-china.com/product/rQBYjXzPHLUS/China-CAS-141-53-7-Hcoona-Chemicals-Organic-Salt-Sodium-Formate.html?pv_id=1ibdvkkf0931&faw_id=1ibdvlslg90b>

[28] Industrial grade formic acid. <https://baoqu888.en.made-in-china.com/product/zJeUYajoIPRG/China-CAS-64-18-6-Liquid-CH2o2-Formic-Acid-Leather-Lowest-Price-85-64-18-6-94-Methanoic-Formic-Acid-Industrial-Grade.html?pv_id=1ibdvp9a0e63&faw_id=1ibdvpu2g0d0>
